# Optical Genome Mapping Identifies Novel Recurrent Structural Alterations in Childhood *ETV6::RUNX1+* and High Hyperdiploid Acute Lymphoblastic Leukemia

**DOI:** 10.1097/HS9.0000000000000925

**Published:** 2023-07-17

**Authors:** Danielle Brandes, Layal Yasin, Karin Nebral, Jana Ebler, Dagmar Schinnerl, Daniel Picard, Anke K. Bergmann, Jubayer Alam, Stefan Köhrer, Oskar A. Haas, Andishe Attarbaschi, Tobias Marschall, Martin Stanulla, Arndt Borkhardt, Triantafyllia Brozou, Ute Fischer, Rabea Wagener

**Affiliations:** 1 Pediatric Oncology, Hematology and Clinical Immunology, Medical Faculty, Heinrich-Heine University and University Hospital Dusseldorf, Germany; 2 Dusseldorf School of Oncology (DSO), Medical Faculty, Heinrich-Heine University, Dusseldorf, Germany; 3 Labdia Labordiagnostik, Clinical Genetics, Vienna, Austria; 4 St. Anna Children´s Cancer Research Institute (CCRI), Vienna, Austria; 5 Institute for Medical Biometry and Bioinformatics, Medical Faculty, Heinrich-Heine University, Dusseldorf, Germany; 6 Center for Digital Medicine, Heinrich-Heine University, Dusseldorf, Germany; 7 Institute of Human Genetics, Hannover Medical School (MHH), Hannover, Germany; 8 St. Anna Children’s Hospital, Department of Pediatric Hematology/Oncology, Pediatric Clinic, Medical University, Vienna, Austria; 9 Pediatric Hematology and Oncology, Hannover Medical School (MHH), Hannover, Germany; 10 German Cancer Consortium (DKTK), partner site Essen/Dusseldorf, Germany

## Abstract

The mutational landscape of B-cell precursor acute lymphoblastic leukemia (BCP-ALL), the most common pediatric cancer, is not fully described partially because commonly applied short-read next generation sequencing has a limited ability to identify structural variations. By combining comprehensive analysis of structural variants (SVs), single-nucleotide variants (SNVs), and small insertions-deletions, new subtype-defining and therapeutic targets may be detected. We analyzed the landscape of somatic alterations in 60 pediatric patients diagnosed with the most common BCP-ALL subtypes, *ETV6::RUNX1*+ and classical hyperdiploid (HD), using conventional cytogenetics, single nucleotide polymorphism (SNP) array, whole exome sequencing (WES), and the novel optical genome mapping (OGM) technique. Ninety-five percent of SVs detected by cytogenetics and SNP-array were verified by OGM. OGM detected an additional 677 SVs not identified using the conventional methods, including (subclonal) *IKZF1* deletions. Based on OGM, *ETV6::RUNX1*+ BCP-ALL harbored 2.7 times more SVs than HD BCP-ALL, mainly focal deletions. Besides SVs in known leukemia development genes (*ETV6*, *PAX5*, *BTG1, CDKN2A*), we identified 19 novel recurrently altered regions (in n ≥ 3) including 9p21.3 (*FOCAD/HACD4*), 8p11.21 (*IKBKB*), 1p34.3 (*ZMYM1*), 4q24 (*MANBA*), 8p23.1 (*MSRA*), and 10p14 (*SFMBT2*), as well as *ETV6::RUNX1+* subtype-specific SVs (12p13.1 (*GPRC5A*), 12q24.21 (*MED13L*), 18q11.2 (*MIB1*), 20q11.22 (*NCOA6*)). We detected 3 novel fusion genes (*SFMBT2::DGKD, PDS5B::STAG2,* and *TDRD5::LPCAT2*), for which the sequence and expression were validated by long-read and whole transcriptome sequencing, respectively. OGM and WES identified double hits of SVs and SNVs (*ETV6*, *BTG1*, *STAG2*, *MANBA*, *TBL1XR1*, *NSD2*) in the same patient demonstrating the power of the combined approach to define the landscape of genomic alterations in BCP-ALL.

## Graphical abstract

**Figure:**
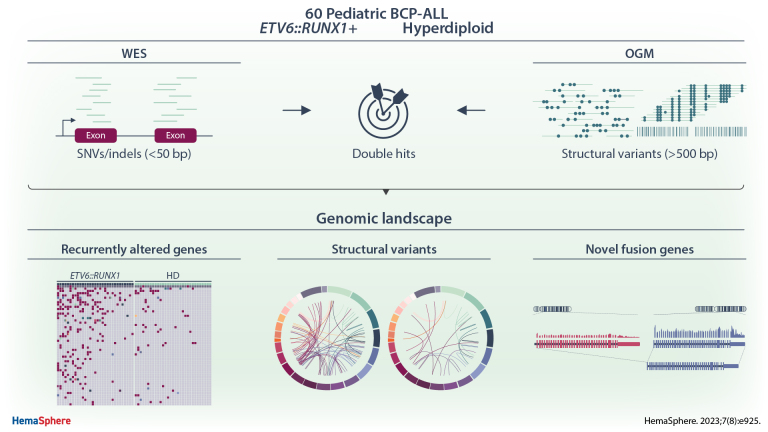

## INTRODUCTION

In the early 1960s and 1970s, a plethora of specific chromosomal abnormalities were identified in B-cell precursor acute lymphoblastic leukemia (BCP-ALL), the most common childhood cancer. Their association with clinical variables, response to chemotherapy, and ultimately outcome has deeply impacted trial design, patient stratification, and therapy allocation. Today, in most routine diagnostic laboratories, a combination of standard, low-cost, and easy-to-perform technologies such as cytogenetics as well as fluorescence in situ hybridization (FISH) and more sophisticated genome-wide analytical tools such as single nucleotide polymorphism (SNP) array analysis are used to determine the molecular genetic make-up of BCP-ALL. The 2 most frequent genetic subtypes, encompassing 50%–55% of all childhood BCP-ALL cases are *ETV6::RUNX1*-translocated (t[12;21][p13;q22]) BCP-ALL and classical hyperdiploid (HD) BCP-ALL, harboring about 51–67 chromosomes per leukemic cell and comprise heterozygous di-, tri-, and tetrasomies.^1^ Both genetic entities usually have excellent therapy responses. Their good outcomes may identify them as candidates for therapy reduction^2^; however, ≈20% of patients experience a relapse, partly with unknown reason, in a subset of initially diagnosed standard-risk patients.^3^ It is not yet known whether additional secondary genomic alterations in *ETV6::RUNX1+* and HD BCP-ALL, particularly the frequently overlooked large-scale structural variations, may help to characterize and subdefine these entities and their outcome even further. For instance, studies by Enshaei et al^4^ and others highlighted that specific copy number (CN) patterns of HD leukemia may influence prognosis and enable further subclassification in low and poor-risk groups.^1^

According to the 2-step model of childhood leukemogenesis, both lesions are acquired in utero during fetal hematopoiesis, and postnatally acquired secondary genetic hits are required for the development of overt disease.^5^ Thus, a distinct constellation of secondary structural variants (SVs) and single-nucleotide variants (SNVs) together with the entity-defining key lesion likely drive the leukemic process.

Most of the mutational landscape of BCP-ALL has been analyzed by conventional cytogenetic methods, SNP or array comparative genomic hybridization and whole genome sequencing (WGS). However, it should be noted that large SVs are particularly difficult to detect by short-read DNA-sequencing methods.^6,7^ This can be attributed to the fact that SVs are likely enriched within or near repetitive DNA regions.^8^ Repetitive DNA is known to be difficult to analyze by short-read sequencing technologies because the resulting reads map ambiguously to multiple locations.^9,10^ The recently introduced genome-wide optical mapping technology analyzes long linear DNA molecules (>150 kb) and covers more repetitive and complicated regions, which allows for the detection of SVs, including inversions, insertions, deletions and translocations, with a size cut-off of 500 bp in length for de novo assembled genomes. The applicability of this approach has recently been shown for various hematological malignancies, and some researchers even advocate for further replacing the existing routine diagnostics methods of those neoplasms with optical genome mapping (OGM).^11–17^

Here, we present a study focusing on 60 HD and *ETV6::RUNX1+* BCP-ALL cases analyzed by whole exome sequencing (WES) and OGM to decipher the landscape of genomic alterations in both entities, with a special focus on their SVs. We report several novel recurrently altered genes, which are not described in previous studies that applied SNP-arrays or WGS.^3,18–20^ Moreover, we demonstrate that the overall genomic landscape of secondary alterations in these 2 types of BCP-ALL is profoundly different.

## MATERIALS AND METHODS

### Patient cohort

This study includes 60 pediatric patients diagnosed with either *ETV6::RUNX1+* or classical HD BCP-ALL in 3 different clinical institutions (Suppl. Tables S1). Patients were enrolled in this study after informed consent was obtained following institutional guidelines in accordance with the declaration of Helsinki. We collected fresh-frozen bone marrow mononuclear cells (BMMNCs) or peripheral blood mononuclear cells (PBMNCs) of all leukemia samples from the time point of initial diagnosis, as well as fibroblasts (n = 16 cases) or fresh-frozen BMMNCs/PBMNCs from remission (n = 44 cases) as matched non-tumor controls. Four of the herein analyzed patients developed a relapse. From 1 *ETV6*::*RUNX1*+ patient, we were able to analyze initial tumor, matched non-tumor, and the relapsed leukemia.

### Cytogenetics and molecular genetics

Karyotypes were determined by chromosomal G-banding. The results of the analysis for *ETV6*::*RUNX1* translocation by FISH of the leukemia samples obtained during routine diagnostics were compiled from the patient records for this study (Suppl. Tables S2 and S3). To detect CN alterations and CN neutral loss of heterozygosity (CNN-LOH), all 60 leukemia samples were analyzed using SNP-arrays. For 18 leukemia cases, the CytoScan HD array (Thermo Fisher Scientific, Waltham, MA) was used as described.^21^ The data were initially aligned to GRCh37 and a lift-over to GRCh38 was performed for downstream analysis. The remaining 42 leukemia cases were analyzed using the Illumina CytoSNP-12 v2.1 array (Illumina, San Diego, CA) according to the manufacturer’s protocol. The array was scanned using the scanner option on the NextSeq550 sequencing platform (Illumina). The raw data were processed and analyzed using the Beeline 2.0.3.3 and the BlueFuse Multi 4.5 software from Illumina. The human reference genome GRCh38 was used. We included SVs encompassing ≥20 probes, ≥50 kb for deletions and duplications, or ≥5 Mb for CNN-LOH. For all samples, B- and T-cell receptor rearrangements were excluded from downstream analysis and SVs overlapping with centromere or reference gap regions. The filtered molecular genetic results are summarized in Suppl. Table S4.

### Whole exome sequencing

WES data was collected from 58 tumor samples and the corresponding non-leukemia, which allows for the detection of somatically acquired alterations. Unfortunately, no sufficient material was available for 2 leukemia samples. WES was performed using the SureSelect Human All Exon V5+UTR kit, All Exon V7 or All Exon V8 (Agilent). The library was paired-end sequenced on a NextSeq550 (2 × 150 bp) sequencer. An average on-target coverage of ≥70× for germline and ≥100× for tumor samples (Suppl. Figure S1) was generated. Refer to Suppl. Materials and Methods I for a detailed description of the bioinformatic processing of WES data. Data of filtered variants are summarized in Suppl. Table S5.

### Optical genome mapping

#### Extraction of high-molecular-weight DNA, labeling, and chip loading

We extracted high-molecular-weight (HMW) DNA from PBMNC or BMMNC at initial diagnosis using the SP Blood and Cell Culture DNA Isolation Kit, according to the manufacturer’s guidelines (Bionano Genomics, San Diego, CA). In addition, HMW DNA was extracted from remission material or fibroblasts (if available) of the respective children as matching nontumor control. Labeling of HMW DNA was performed using the DLS DNA Labeling Kit (Bionano Genomics), according to the manufacturer’s protocol (Bionano Prep Direct Label and Stain [DLS] Protocol 30206). The labeled DNA was loaded on a Saphyr G2.3 chip and molecules were imaged using the Saphyr instrument (Gen 2, Bionano Genomics). Overall, we performed OGM on 60 matched tumor/non-tumor pairs (120 samples), and 1 sequential sample including tumor material from initial diagnosis and relapse in comparison to matched non-tumor material (ALL4). Refer to Suppl. Figure S2 for an overview of the applied OGM workflow.

#### Data collection and analysis

We collected an average of 124× effective coverage for all tested non-tumor samples (420 GB/sample) and 384× for tumor samples (1300 GB/sample) (Suppl. Figure S1). The Bionano Access Server ([BAS], Bionano Genomics, San Diego, CA) was used for data analysis and visualization. The human reference genome GRCh38 was used for alignment. SVs were called using the de novo (DN) assembly (BAS 1.5.2) and rare variant pipeline (RVP) (BAS 1.7) for each non-tumor and tumor sample. Before the OGM data of the leukemia samples could be processed using the DN pipeline, the molecule files had to be downsampled to 480 GB with a minimal molecule length of 150 kb. To differentiate between somatic and germline SVs, we performed dual annotation for the DN and RVP data. In the ALL4 case, we used the trio annotation module, allowing for the direct comparison of the data from initial diagnosis and relapse as well as filtering for variants detected in the matching non-leukemia controls.

#### Data Filtering and Interpretation

We excluded all SVs that were present in the matching nonleukemia samples, which likely represent germline SVs. SVs were filtered as described in Suppl. Material and Methods II. The curated lists of SVs, including deletions, duplications, insertions, inversions, inter and intrachromosomal translocations, detected by both DN and RVP pipelines were merged. The applied software calls large inversions and deletions as intra-chromosomal translocations. Both DN and RVP pipelines contain an additional CN algorithm tool that detects CN alterations, mainly deletions and duplications ≥5 Mb including aneuploidy. Accordingly, the final dataset included the filtered SVs detected by the DN and RVP algorithms and the SVs ≥5 Mb detected with the CN tool.

For downstream analysis, we differentiated between SVs ≥5 Mb and SVs ≤5 Mb. The latter included inversions and translocations for each case, as well as deletions and duplications ≤5 Mb to delineate focal SVs and detect recurrently altered regions/genes as previously described.^3^ Moreover, we incorporated the detected CNN-LOH identified by SNP-array. Refer to Suppl. Table S6-S8 and Suppl. Figures S3 and S4 for the OGM results.

### Validation of selected SVs by NGS

#### Long-read sequencing

PacBio (Pacific Biosciences, Menlo Park, CA) long-read sequencing was performed on 11 tumor samples where sufficient material was available to validate selected SVs that were newly detected by OGM. HMW DNA (5 µg) was used for the continuous long-read (CLR) workflow (>25 kb size cut-off), according to the manufacturers’ instructions. Each sample was submitted to sequencing on a PacBio Sequel II (performed by Genomics and Transcriptomics Laboratory, BMFZ, Dusseldorf, Germany) using 1 SMRT cell. We generated an average of 31× coverage (~231 GB/sample). Raw bam files were sorted and aligned to the reference genome GRCh38 using pbmm2 (v1.3.0; https://github.com/PacificBiosciences/pbmm2). Sorted bam files were visualized using the Integrative Genomics Viewer (IGV, https://www.broadinstitute.org/igv, version 2.11.9). Refer to Suppl. Materials and Methods III for detailed analysis of the CLR data.

#### RNA sequencing

To validate newly detected gene fusions, data of RNA sequencing (RNA-seq) were collected from 6 tumor samples. Data of 2 samples were generated as described before.^22^ Total RNA of 4 cases was isolated from fresh-frozen cells and submitted for RNA-seq at the Genomics and Proteomics Core Facility (DKFZ, Heidelberg, Germany) or Genomics and Transcriptomics Laboratory (BMFZ, Dusseldorf, Germany). For detailed description of RNA-seq workflow refer to Suppl. Material and Methods IV. Fusions were detected and visualized using Arriba v2.4.0 algorithm.^23^ Arriba was run with default settings against the GRCh38 reference genome with the GENECODE annotation.

## RESULTS

### OGM detects 1.6- to 3-fold more deletions than conventional SNP-arrays in a cohort of 60 primary pediatric BCP-ALL patients

**Figure 1.**
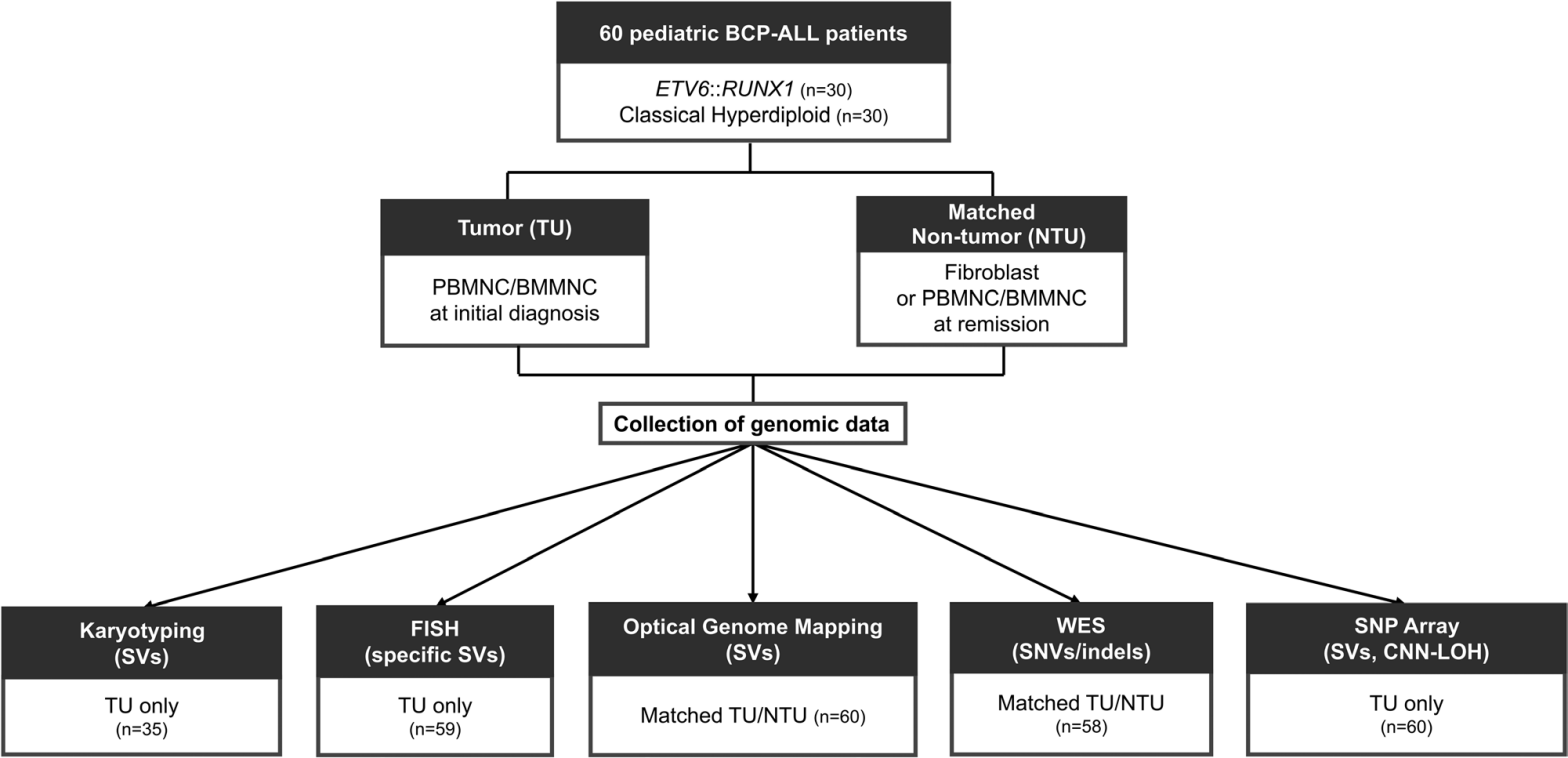
**Overview of genomic data collection from 60 pediatric BCP-ALL patients.** Tumor and matched non-tumor samples from 30 *ETV6*::*RUNX1* and 30 classical HD BCP-ALL patients were analyzed. A comprehensive data set was collected to detect SVs, CNN-LOHs, and SNVs/indels using karyotyping, FISH, OGM, WES, and SNP-array. BCP-ALL = B-cell precursor acute lymphoblastic leukemia; BMMNC = bone marrow mononuclear cells; CNN-LOHs = copy number neutral loss of heterozygosity; FISH = fluorescence in situ hybridization; HD = hyperdiploid; OGM = optical genome mapping; PBMMNC = peripheral blood mononuclear cells; SNP = single nucleotide polymorphism; SV = structural variants; WES = whole exome sequencing.

We studied somatically acquired SNVs, insertions-deletions (indels), and SVs in 60 pediatric patients with *ETV6::RUNX1+* and classical HD BCP-ALL (each n = 30) (Suppl. Table S9). The cohort consisted of 32 male (53%) and 28 (47%) female newly diagnosed patients with a median age of 3.8 years (range, 1.6–17 years). The majority of patients (87%; 52/60) were stratified as standard or medium risk, while 13% (n = 8/60) were classified as high risk. In total, 93.3% (56/60) of the patients remained in complete remission, whereas 6.7% (4/60) relapsed. By analyzing tumor and matched non-tumor samples of these leukemia patients using OGM and WES, we were able to detect somatically acquired alterations potentially associated to the leukemogenesis. A comprehensive set of genomic data was generated by adding diagnostically reported data from conventional cytogenetics (karyotyping and FISH) and SNP-array data (Figure 1).

**Figure 2.**
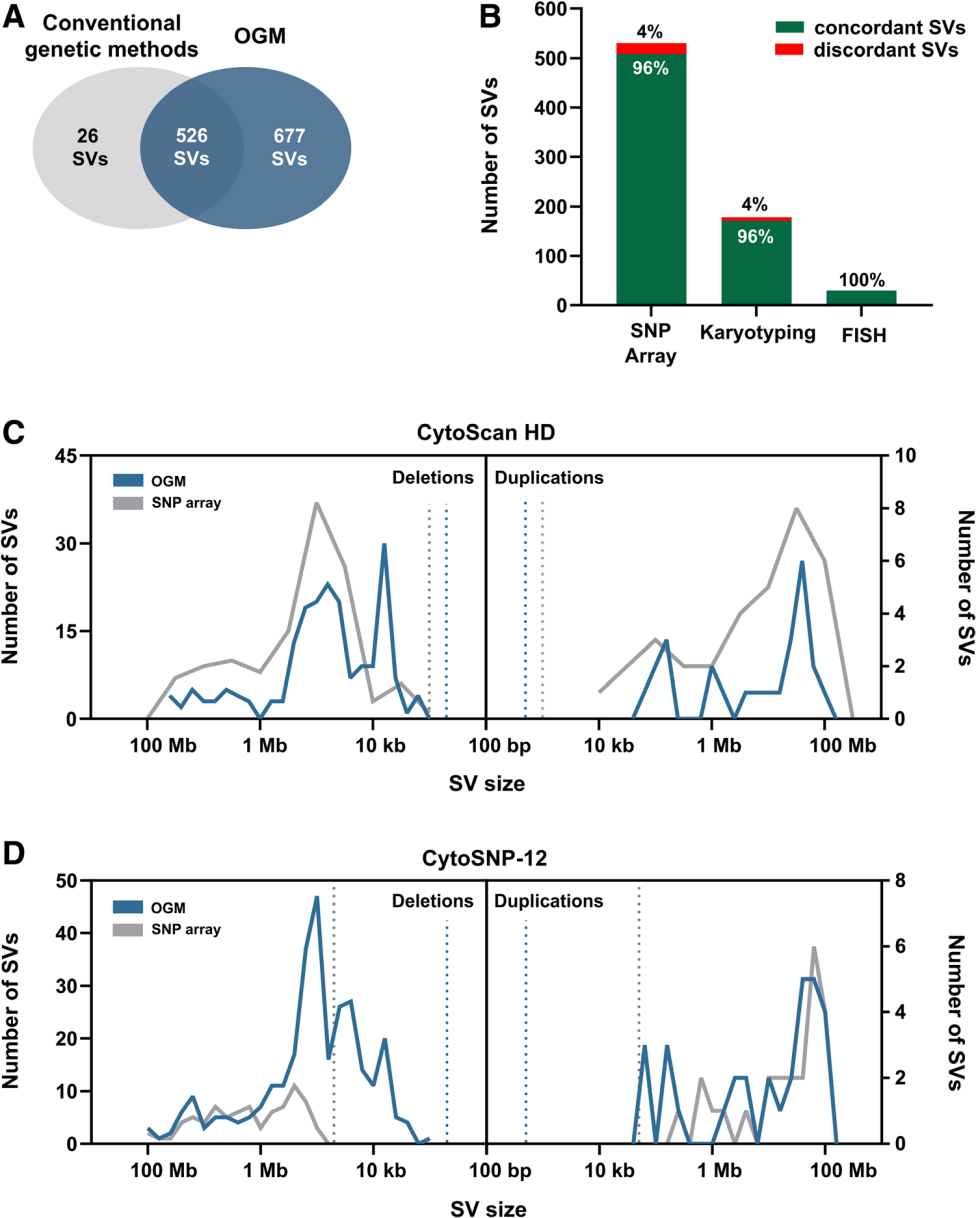
**Comparison of SVs detected by conventional genetic techniques and OGM in 60 primary BCP-ALL cases.** (A) OGM reliably detects 95% (526/552) of SVs detected by conventional genetic methods (SNP-array, karyotyping, and FISH), including all hallmark aberrations such as the *ETV6::RUNX1* translocation and hyperdiploidy. OGM detected 677 additional SVs compared with standard methods. (B) Frequencies of concordant and discordant SVs detected by karyotyping, SNP-array, and FISH compared with OGM. (C) Size distribution of deletions and duplications detected by OGM (green) and CytoScanHD (SNP-array, gray) in 18 cases. Dashed lines indicate the detection limit of 1 kbp (SNP-array, gray) and 500 bp (OGM, green). (D) Size distribution of deletions and duplications detected by OGM (green) and CytoSNP-12 (SNP-array, gray) in 42 cases. Dashed lines indicate the detection limit of 50 kbp (SNP-array, gray) and 500 bp (OGM, green). BCP-ALL = B-cell precursor acute lymphoblastic leukemia; FISH = fluorescence in situ hybridization; HD = hyperdiploid; OGM = optical genome mapping; SNP = single nucleotide polymophism; SV = structural variants.

Performing OGM, we yielded an average coverage of 384× in the BCP-ALL samples and 124× in the non-leukemia samples (Suppl. Figure S1). By applying 2 SV calling pipelines (DN and RVP) together with an integrated CN pipeline, we detected a total of 1203 somatic SVs by OGM. First, we analyzed the concordance rate of SVs detected by conventional genetic techniques and OGM. OGM reliably identified 95% (526/552) of SVs identified by karyotyping, FISH, and/or SNP-array, including all hallmark translocations t(12;21)(p13;q22) and chromosomal gains in HD BCP-ALL (Figure 2A). Notably, OGM resolved 3-way translocations involving t(12;21)(p13;q22) present in 9 of 30 *ETV6::RUNX1* BCP-ALL cases. All aberrations detected by FISH and 96% (164/171) of SVs identified by karyotyping were observed by OGM. Of note, the translocation der(14)t(10;14)(p11;p11) with breakpoints in centromeric regions was not detectable by OGM. In total, 96% (486/508) of SVs identified by SNP-array were detectable by OGM (Figure 2B). Alterations missed by OGM, even after manual reevaluation, included, among others, a gain of chromosome Y, 2 focal mosaic deletions (~30% of cells) in chromosome 13q and 1 mosaic gain in 5p15.33p15.1 (~15% of cells) (Suppl. Table S10).

Next, we compared the lengths of the deletions and duplications detected by OGM and SNP-array. As we used 2 different types of SNP-arrays that have different probe coverages (CytoSNP-12 array ≈300,000 versus CytoScanHD ≈2,670,000 SNPs targeting probes) and detection limits with respect to alteration length, we performed 2 separate analyses for each array (Figure 2C and 2D). Considering the CytoSNP-12 array’s detection limit of 50 kb for the OGM data, the absolute numbers of deletions detected by OGM exceeded the number identified by CytoSNP-12 array by 3-fold (245 versus 81). By contrast, considering a detection limit of 1 kb using CytoScanHD, the number of deletions detected by OGM was only 1.6 times higher compared with the array (197 versus 122 deletions, respectively). Interestingly, the number of somatic duplications detected by OGM and both array types was not notably different (OGM n= 28 versus CytoSNP-12 n = 20 with 50 kb detection limit; OGM n = 25 versus CytoScanHD n = 33 with 1 kb detection limit), likely due to the overall rareness of these events in our data.

Compared with the above-described conventional genetic methods, OGM detected an additional 677 SVs, including complex 3-way translocations (Figure 2A). About 66.3% of those SVs consisted of deletions (449/677) with a median size of 40.5 kb (range, 1 kb to 3.6 Mb). In total, 55.2% (248/449) of the deletions detected by OGM were <50 kb, which is the lower detection limit of the CytoSNP-12 array. The remaining additional SVs found by OGM were insertions (n = 89), inter and intrachromosomal translocations (n = 76 and n = 33, respectively), inversions (n = 17), and duplications (n = 13); 37.2% (252/677) of those SVs were detected by both SV detection algorithms, whereas 53.5% (362/677) were solely identified by the RVP pipeline.

### *ETV6::RUNX1*+ BCP-ALL harbor 2.7-fold more SVs, mainly deletions, compared with HD BCP-ALL as determined by OGM

**Figure 3.**
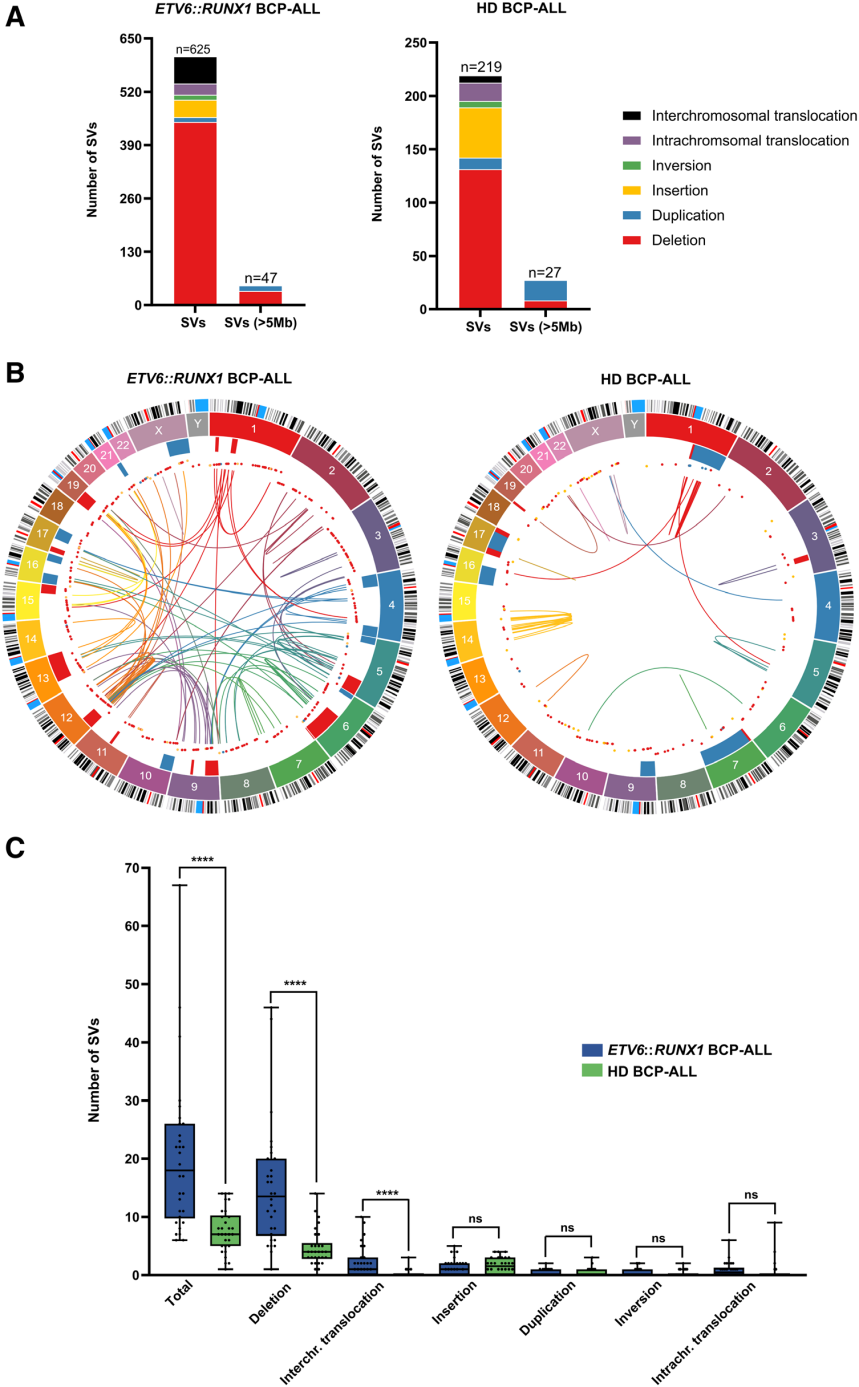
**Frequencies of SVs detected in 60 primary BCP-ALL cases by OGM in addition to their hallmark alterations.** The defining *ETV6::RUNX1* translocation and numerical whole chromosomal alterations are not included. (A) Total numbers of SVs and SVs ≥5 Mb in 60 primary BCP-ALLs. (B) Circos plots showing from the outer to the inner circle: the chromosomal ideogram; the chromosome number; chromosomal regions of deletions (red) and duplications (blue) ≥5 Mb, focal deletions (red), duplications (blue) and insertions (yellow); and intra and interchromosomal translocations and inversions of *ETV6::RUNX1+* (n = 30, left circos plot) and HD (n = 30, right circosplot) BCP-ALL. (C) Box plots depicting the number of SVs per case for all SVs (total) and for each SV type in *ETV6::RUNX1*+ (blue) and HD (green) BCP-ALL subtypes. Box and whiskers indicate median, 25 of and 75% quartiles and ±range. Statistical significance was assessed between the 2 BCP-ALL subtypes using the Mann-Whitney *U* Test. ****P < 0.0001. BCP-ALL = B-cell precursor acute lymphoblastic leukemia; HD = hyperdiploid; ns = not significant; OGM = optical genome mapping; SV = structural variants.

We compared the frequencies of SVs detected by OGM between the 2 genetic subgroups of BCP-ALL (*ETV6::RUNX1+* and HD leukemia, n = 30 each) that occurred in addition to their hallmark events. We identified 2.7 times more somatic SVs (focal deletions and duplications ≤5 Mb, insertions, inversions, inter and intrachromosomal translocations) in *ETV6::RUNX1+* compared with HD BCP-ALL (n = 625 versus n = 219 SVs, respectively) (Figure 3A and 3B). SVs ≥5 Mb were twice as frequent in *ETV6::RUNX1+* leukemia (n = 47 versus n = 27) (Figure 3A and 3B; Suppl. Table S6). In both subtypes, 68% (577/844) of all SVs ≤5 Mb, the most prominent type of SV in both subtypes, were focal deletions. Focal deletions were 3.4-fold more frequent in *ETV6::RUNX1*+ BCP-ALL compared with HD BCP-ALL (n = 446 versus n = 131 focal deletions). The burden of deletions in *ETV6*::*RUNX1* BCP-ALL was 13.5 deletions (median; range, 1–46) per tumor compared with 4 deletions (median; range, 1–14) in HD cases (Figure 3C; Suppl. Table S7).

We observed secondary interchromosomal translocations in 20 *ETV6::RUNX1* BCP-ALL cases that occurred in addition to their hallmark translocation. By contrast, secondary interchromosomal translocations were rarely detected in HD BCP-ALL cases (7 translocations in 5 cases).

**Figure 4.**
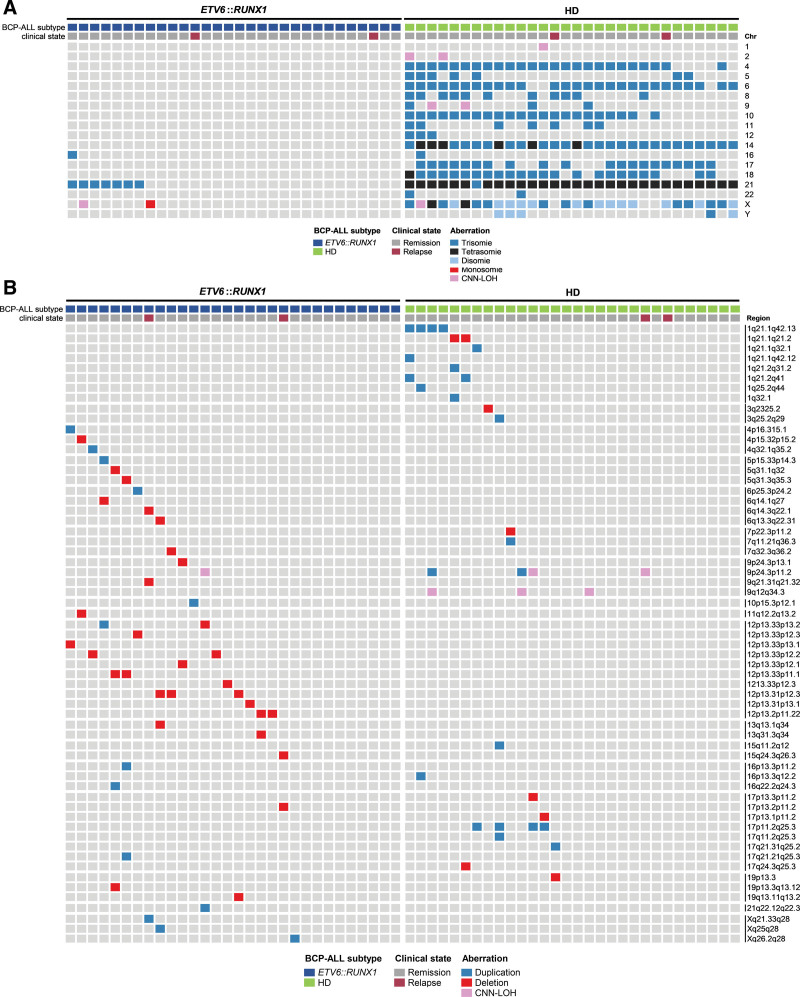
**Landscape of aneuploidy and SVs ≥5 Mb in *ETV6::RUNX1+* and HD BCP-ALL identified by OGM and SNP-array.** Depicted are (A) aneuploidies and whole chromosomal CNN-LOHs and (B) SVs ≥5 Mb and CNN-LOHs not affecting the whole chromosome, identified *ETV6::RUNX1*+ and HD BCP-ALL cases. Each column represents a case and each line an altered region. The color code indicates the type of alteration detected. BCP-ALL = B-cell precursor acute lymphoblastic leukemia; CNN-LOHs = copy number neutral loss of heterozygosity; HD = hyperdiploid; OGM = optical genome mapping; SNP = single nucleotide polymorphism; SV = structural variants.

We further compared patterns of aneuploidy events and SVs ≥5 Mb including chromosomal p/q alterations (detected by OGM), as well as CNN-LOH (observed by SNP-array) between both genetic subgroups. In terms of aneuploidy events, we detected 7 *ETV6::RUNX1+* cases harboring a trisomy 21, 1 case with trisomy 16, and 1 female case with monosomy X (Figure 4A). Whole chromosomal CNN-LOH were detected in only 1 *ETV6::RUNX1+* case on chrX (Figure 4A). In classical HD BCP-ALL, all cases showed typical aneuploidy patterns including tetrasomy 21 (29/30); except 1 patient with trisomy 21; trisomy 14 (24/30) or tetrasomy 14 (6/30) (Figure 4A). Whole chromosomal CNN-LOH were identified in chr2 and chr9 in 2 HD BCP-ALL and in chr1 and chrX in 2 different HD BCP-ALL (Figure 4A). Of note, to define additional potential risk factors in HD BCP-ALL as described in Enshaei et al,^4^ we analyzed OGM defined aneuploidy patterns. Specifically, HD BCP-leukemia without trisomy 17 and 18 or harboring either trisomy 17 or 18 with trisomy 5 or 20 are associated with poor risk. We detected 6 poor-risk HD leukemia (ALL31, ALL41, ALL49, ALL51, ALL52, and ALL55), based on these criteria.

Large-scale SVs ≥5 Mb (not affecting the whole chromosome) detected by OGM encompassed deletions and duplications (Figure 4B). Large-scale deletions were more frequent in *ETV6::RUNX1+* than in HD BCP-ALL (n = 31 versus n = 8, respectively), including the secondary large-scale deletion (mean of 17 Mb) in chr12p that led to the loss of the second *ETV6* allele. By contrast, only 1 deletion on 1q21.1q21.2 was found to be recurrently affected in 2 of 30 HD BCP-ALL cases. Large duplications were more frequent in HD than in *ETV6*::*RUNX*1+ BCP-ALL (n = 20 versus n = 13, respectively). Recurrently affected regions were identified on chromosome 1q21.1q42.13 (n = 4/30, HD BCP-ALL) and 17p11.2q25.3 (n = 4/30; HD BCP-ALL). In both BCP-ALL entities, CNN-LOH on chromosome arms 9p (n = 3) and 9q (n = 3) was detected.

Moreover, we were able to analyze sequential tumor samples in 1 *ETV6::RUNX1* BCP-ALL case (ALL4) who relapsed 48 months after initial diagnosis. Comparing the diagnostic and the relapse samples in terms of common and acquired SVs detected by OGM, we observed 34 shared SVs, 34 acquired SVs, and 11 SVs that were lost in the relapse sample (Suppl. Figure S5). Acquired SVs included an inv(X)(p11.3;q13.2) potentially leading to a *KDM6A::XIST* gene fusion (Suppl. Figure S5B; Suppl. Table S8).

### The combination of OGM and WES revealed novel commonly recurrent as well as subtype-specific altered regions and genes in *ETV6::RUNX1*+ and HD BCP-ALL

**Figure 5.**
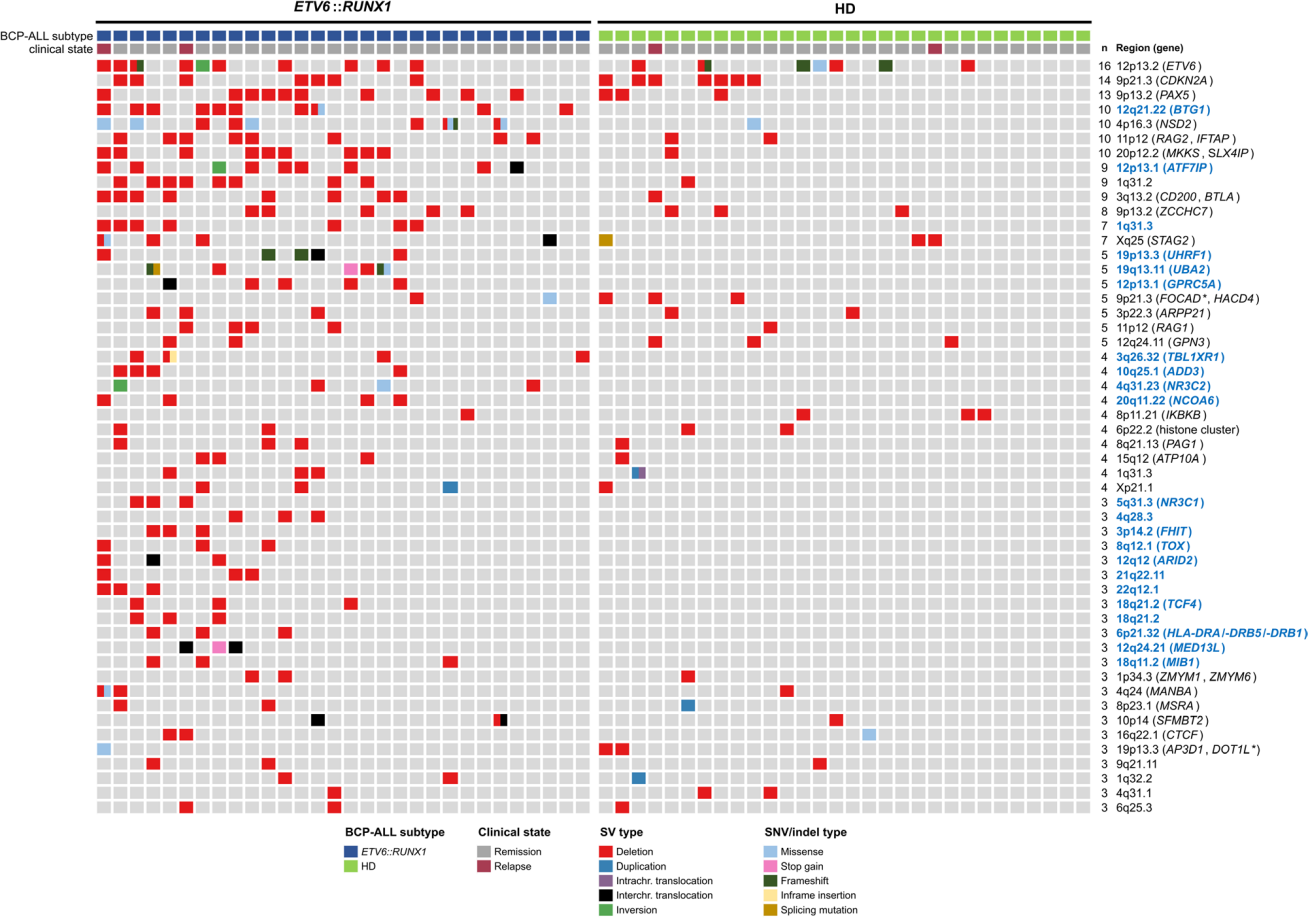
**Combination of OGM and short-read whole exome sequencing reveals recurrently altered regions and genes in 60 primary BCP-ALL cases.** The oncoprint depicting the combined dataset of MARs by SVs identified by OGM and SNVs/indels affecting the potential target gene, which are recurrently affected in n≥3 BCP-ALL (5%). Every column indicates a case and each line a recurrently altered region. The color code indicates the type of SV and SNV/indel alteration. The number of cases (n) with the specific region of the MARs is given on the right. Subtype-specific alterations are highlighted in blue for *ETV6::RUNX1+* BCP-ALL. If 2 or more potential target genes are located in the MAR, an asterisk (*) indicates the gene affected by SNV/indel. BCP-ALL = B-cell precursor acute lymphoblastic leukemia; HD = hyperdiploid; MARs = minimal altered regions; OGM = optical genome mapping; SV = structural variant.

Our next aim was to identify recurrently altered regions and genes affected by either SVs ≤5 Mb and/or SNVs/indels, combining both OGM and WES data sets. We determined the minimal altered regions (MARs; not differentiating between the SV types) and the therein located potential target genes detected in ≥3 BCP-ALL cases by OGM. Next, we checked whether target genes located within these MARs were affected by SNVs/indels in the BCP-ALL cohort, that is allowing for the detection of cases with double hits (SVs and SNV/indel) or single hits harboring either an SV or SNV/indel in a target gene (Figure 5).

Overall, we detected 52 MARs, including 40 MARs harboring a potential target gene and 12 MARs, which were located in intergenic regions. We identified more *ETV6::RUNX1+* cases with MARs compared with classical HD BCP-ALL cases (n = 30 versus n = 24, respectively). The most frequently detected MARs and therein located potential target genes, which were observed in ≥10% of both BCP-ALL subtypes, were 12p13.2/*ETV6* (27%; 16/60), 9p21.3/*CDKN2A* (23%; 14/60), 9p13.2/*PAX5* (22%; 13/60), 4p16.3/*NSD2* (17%; 10/60), 11p12/*RAG2*/*IFTAP* (17%; 10/60), 20p12.2/*MKKS*/*SLX4IP* (17%; 10/60), 3q13.2/*CD200*/*BTLA* (15%; 9/60), 9p13.2/*ZCCHC7* (13%; 8/60), and Xq25/*STAG2* (12%; 7/60). Less frequently detected, but recurrently altered regions/genes (n=2; 3%) in both *ETV6::RUNX1+* and HD BCP-ALL included focal deletions affecting, for example 7p12.2/*IKZF1* and 3q25.1/*MBNL1* (Suppl. Figure S6).

We identified 11 novel, not yet reported to the best of our knowledge, MARs affecting 8 potential target genes that are commonly affected in both entities (Suppl. Table S11). Hence, they might be involved in general BCP-ALL leukemogenesis. These MARs and potential target genes included 9p21.3/*FOCAD/HACD4* (8%; 5/60), 8p11.21/*IKBKB* (7%; 4/60), 1p34.3/*ZMYM1/6* (5%; 3/60), 4q24/*MANBA* (5%; 3/60), 8p23.1/*MSRA* (5%; 3/60), and 10p14/*SFMBT2* (5%; 3/60). We validated selected SVs in tumors where sufficient material was available for long-read sequencing and with the available SNP-array data (Suppl. Table S11; Suppl. Figure S7).

**Figure 6.**
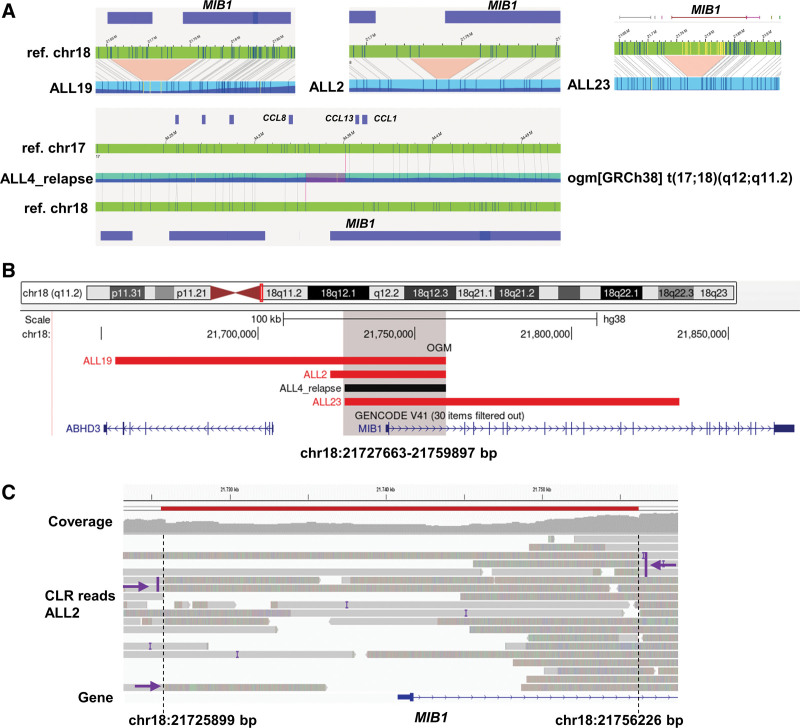
**Examples of herein novel recurrently altered region affecting *MIB1* as potential target gene in ETV6::RUNX1 BCP-ALL detected by OGM.** (A) Optical maps (blue) of 4 ETV6::RUNX1 BCP-ALLs (ALL19, ALL2, ALL23, ALL4_relapse) aligning to reference map of chromosome 18 (green) are shown. Top: Red highlights indicate deleted regions overlapping with the *MIB1* gene locus in 3 BCP-ALL cases. Bottom: t(17;18)(q12;q11.2) occurring in a relapsed leukemia with potential breakpoint in *MIB1* (indicated by pink line) is shown. (B) Detailed chromosomal locations of the SVs affecting the *MIB1* gene locus of four BCP-ALL are shown in the UCSC genome browser. Red bars indicate potentially deleted regions. Black bar shows region of the potential translocation breakpoint. Minimal altered region on 18q11.2 (chr18:21,727,663–21,759,897 bp) is highlighted in gray and overlaps with promotor region of *MIB1*. (C) CLR sequencing of ALL2 validate the deletion (chr18:21,725,899–21,756,226 bp) of the *MIB1* promotor region. CLR reads of the *MIB1* locus are shown (gray bars). Dashed lines indicate breakpoints of the deletion. Split reads supporting the deletion are marked by purple arrows. Decreased coverage of aligned reads indicates a deletion (red bar). Dashed lines indicate breakpoints of the deletion. Split reads supporting the deletion are marked by purple arrows. Decreased coverage of aligned reads indicates a deletion (red bar). BCP-ALL = B-cell precursor acute lymphoblastic leukemia; CLR = continuous long-read; OGM = optical genome mapping; SV = structural variants.

Moreover, 22 *ETV6::RUNX1+* subtype-specific MARs (22/54) have been identified, of which 17 could be associated with potential target genes. Six of those genes were also affected by 1 or several SNVs/indels. Four of them have previously been described to be recurrently affected in this subtype: *BTG1* (33%; 10/30), *ATF7IP* (30%; 9/30), UH*RF1* (17%; 5/30), and *UBA2* (17%; 5/30).^3,19,20^ Applying long-read sequencing, we validated a deletion in ALL4 (chr19:4,804,231-4,918,731 bp) by which the promotor region of *UHRF1* was removed (Suppl. Figure S7D) and a deletion in ALL7 (chr19:34,458,211–34,460,111 bp), which removed exon 13 of *UBA2* (Suppl. Figure S7H). Strikingly, we herein identified 4 novel, that is, not yet reported to the best of our knowledge, potential target genes in the MARs that are specifically and recurrently altered in the *ETV6::RUNX1+* BCP-ALL subgroup by SV and/or SNV (Suppl. Table S11). These included 12p13.1/*GPRC5A* (17%; 5/30), 20q11.22/*NCOA6* (13%; 4/30), 12q24.21/*MED13L* (10%; 3/30), and 18q11.2/*MIB1* (10%; 3/30). We validated selected SVs affecting a novel target gene in tumors where sufficient material was available by long-read sequencing (Suppl. Table S11; Figure 6; Suppl. Figure S7). The MAR on 18q11.2 was defined by 3 deletions detected by OGM, which affected the promotor region of *MIB1* and the first coding exon of the gene (Figure 6A and 6B). The analysis of the *MIB1* region in the existing long-read sequencing data for this case showed that the promotor region was deletion (chr18:21,725,899–21,756,226 bp) in ALL2 (Figure 6C). Interestingly, we also detected a somatically acquired translocation t(17;18)(q12;q11.2) in the relapsed leukemia of case ALL4, with one of the breakpoints being located in the MAR of the *MIB1* gene locus (Figure 6A; Suppl. Table S8).

By combining OGM and WES datasets, we were able to identify double hits in genes targeted by SVs and SNVs/indels. The combination of deletions and truncating SNVs/indels is of particular interest, as this might indicate the presence of a tumor-suppressor gene. Mining our combined dataset, we identified 6 genes that carried such double hits. The *ETV*6 gene was affected by secondary deletions and frameshift mutations in 1 *ETV6::RUNX1+* and 1 HD BCP-ALL case. Likewise, *NSD2* was affected by a deletion and a frameshift mutation in 1 *ETV6::RUNX1* BCP-ALL case while another carried a deletion as well as frameshift and missense mutations. Further double hits of deletions and concomitant SNVs were observed in *BTG1*, *STAG2*, *MANBA*, and *TBL1XR1* in 5 *ETV6::RUNX1+* BCP-ALL cases.

### Mutational landscape of *ETV6::RUNX1*+ and classical HD BCP-ALL as assessed by WES

We analyzed the mutational landscape of the 2 BCP-ALL subgroups, considering only the SNVs/indels as detected by short-read WES. In *ETV6::RUNX1+* BCP-ALL, we identified a median of 18 SNVs/indels (range, 2–198) per case compared with 13.5 SNVs/indels (range, 5–35) per HD BCP-ALL case (ns, *P* = 0.057, Mann-Whitney test) (Suppl. Figure S8).

For the most frequently mutated genes (n≥3), we classified the recurrent mutations with regard to probable oncogenicity (Suppl. Table S12; Suppl. Materials and Methods I). The most frequently mutated genes in both subtypes were *NRAS* (24%; 14/58), *NSD2* (10%; 6/58), and *ETV6* (9%; 5/58) (Suppl. Figure S9). The herein detected *NRAS* mutations affected the well-known hotspot codons located in the GTPase domain. As previously reported,^3,24^ these mutations were more prevalent in HD than in *ETV6::RUNX1+* BCP-ALL cases (40% versus 7%) (Suppl. Figure S9; Suppl. Table S12).

Moreover, we identified recurrent *ETV6::RUNX1+* subtype-specific SNVs/indels in *CCDC168* (11%; 3/28) and *UBA2* (11%; 3/28), whereas in the HD BCP-ALL group, subtype-specific recurrent somatic SNVs/indels were observed in *KRAS* (27%; 8/30), *CREBBP* (20%; 6/30), *FLT3* (17%; 5/30), and *PTPN11* (10%; 3/30).

### Detection of poor prognostic markers including *TP53* and *IKZF1* deletions by OGM

We examined, whether there would be an additional benefit of applying OGM alongside standard techniques in order to detect prognostic genomic alterations in BCP-ALL. Accordingly, we mined our OGM data for SVs, which have been described to be relevant to prognosis.^3^

In *ETV6::RUNX1+* BCP-ALL case ALL4, which was stratified as a standard-risk case, but later developed a relapse, a somatic loss of 12 Mb length encompassing the *TP53* gene locus was detected (chr17:5,423,849–18,312,173 bp) (Suppl. Table S6). In line with this, case ALL4 had a mutator phenotype harboring 198 SNVs at initial diagnosis, which represents an excessive number of mutations compared with other *ETV6*::*RUNX1* cases (median 18 SNVs/indels [range, 2–60]). However, this *TP53* deletion was also present in the SNP-array data, which were retrospectively gathered in the framework of this study (Suppl. Table S4). We identified 2 double hits (combination of SV and SNV) in the genes *STAG2* and *MANBA* that were already present at initial diagnosis (Suppl. Tables S5 and S7). The 2 deletions affecting *STAG2* (chrX:123,968,231–124,001,631 bp) and *MANBA* (chr4:102,682,958–102,825,431 bp) were validated by long-read sequencing (Suppl. Figure S7A and S7E).

We could retrospectively show that *ETV6::RUNX1+* BCP-ALL case ALL13 harbored an intragenic *IKZF1* deletion. Patients characterized by *IKZF1* deletions that co-occur with deletions in *CDKN2A/B*, *PAX5*, or *PAR1* in the absence of *ERG* deletions belong to a recently described poor prognostic group, *IKZF1*^plus^, with lower event-free survival and higher rates of relapse in an MRD-dependent manner.^25^ ALL13 carried a 39 kb deletion (chr7:50,306,321–50,348,483 bp), which led to loss of exon 2 and 3 within the *IKZF1* gene and a concomitant 61 kb deletion (chr9:36,841,199–36,916,988 bp) affecting exon 8 and 9 of the *PAX5* gene. Deletion in *IKZF1* (chr7:50,308,978–50,348,439 bp) and *PAX5* (chr9:36,843,016–36,903,796 bp) were confirmed by long-read sequencing (Suppl. Figure S10). This case would have fulfilled the *IKZF1*^*plus*^ criteria, although these criteria are not clinically applied to *ETV6::RUNX1* positive cases. Neither deletion was detectable via the CytoSNP-12 v2.1 array, underscoring the benefits of applying OGM.

We detected another *IKZF1* alteration, an 88 kb deletion removing exon 2-7 (chr7:50,306,321–50,399,656 bp), which was identified by OGM and not detected by the CytoScanHD-array in an HD BCP-ALL (ALL55). OGM data suggested a low variant allele frequency (~0.06) of the *IKZF1* deletion, which is under the detection limit of the applied array. The prognostic impact of subclonal *IKZF1* deletions is still unclear and needs to be assessed.^26^ No other deletion of the *IKZF1*^*plus*^ criteria occurred in this medium risk patient.

Our results show that OGM is particularly useful for the detection of SVs within the highly repetitive region of the *IKZF1* gene, where array platforms lack probe coverage.

### Novel secondary translocations and potential fusion genes were frequently detected in *ETV6::RUNX1*+ BCP-ALL

One of the strengths of OGM is the ability to detect interchromosomal translocations and complex rearrangements. We mined our data for the presence of potential secondary translocations that might lead to the genesis of novel fusion genes. Notably, due to the resolution of OGM, the exact breakpoints of the translocation could not be estimated and hence, the fusions are approximations. We identified 10 translocations where gene fusions potentially led to disruption of the juxtaposed genes, including recurrent translocations affecting the *MED13L* gene locus on 12q24.21 in 2 *ETV6::RUNX1+* BCP-ALL cases. In ALL13, a t(6;12)(p21;q24) was reported by karyotyping during routine diagnostics. By OGM, this translocation was determined to be a complex 3-way translocation t(6;8;12)(p12.1;q24.23;q24.21), which might lead to the disruption of *MED13L* and *HMGCLL1* (Suppl. Figure S11). The disruption of *MED13L* by the translocation was verified by long-read sequencing (Suppl. Figure S7M).

In this study, 9 translocations detected by OGM, which have not yet been described in the Mitelman database or by Brady et al,^3^ might lead to the in-frame fusion of 2 genes. Seven of those translocations were detected in *ETV6::RUNX1+* and 2 in HD BCP-ALL cases (Suppl. Table S13) and are further described below.

**Figure 7.**
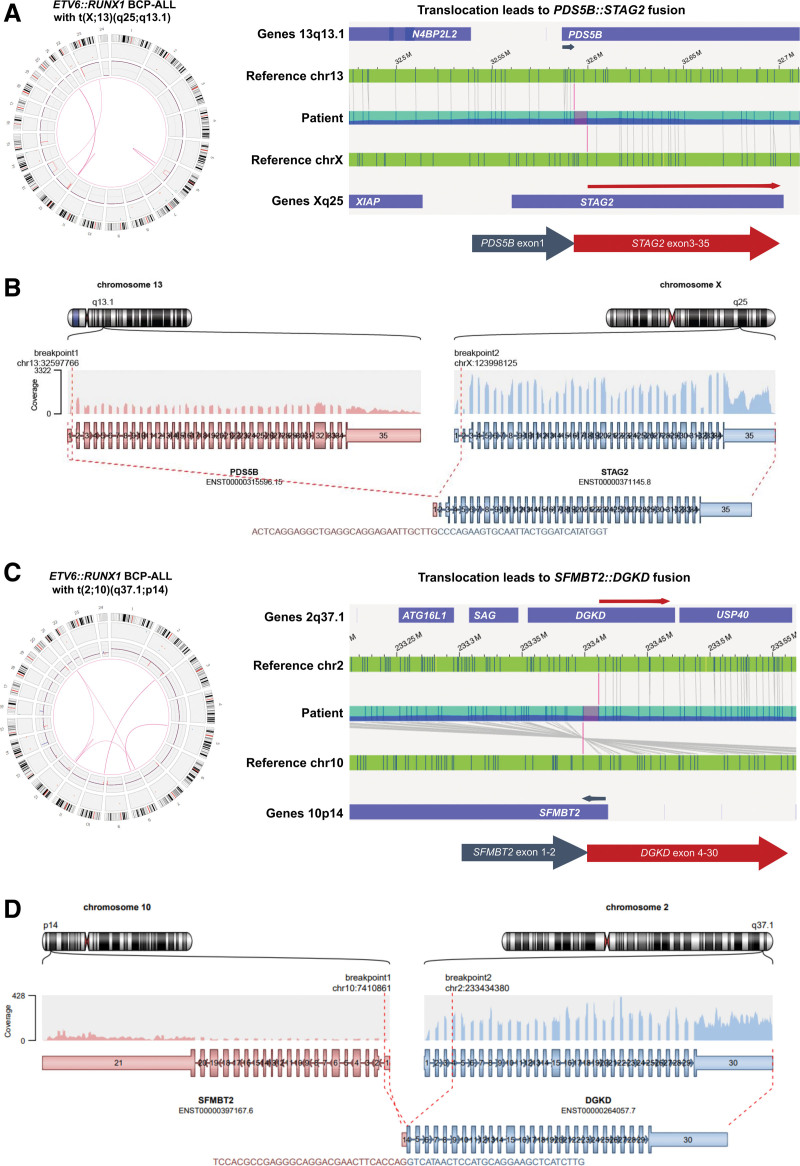
**OGM and RNA-seq reveal expression of novel fusion genes in *ETV6::RUNX1* BCP-ALL.** (A) Left: Circos plot showing somatic SVs of ALL28 with secondary translocation t(X;13)(q25;q13.1) detected by OGM (left). Right: Detailed view of patient optical map (blue) partly aligning to reference chromosomes (green) 13 and X, indicating a translocation that leads to potential fusion of *PDS5B* exon1 (gray arrow) to *STAG2* exon 3–35 (red arrow). Potential breakpoints are indicated in pink. (B) Validation of *PDS5B::STAG2* expression in ALL28 by RNA-seq. Arriba output of RNA-sequencing data is shown indicating a t(X;13)(q25;q13.1) that leads to in-frame fusion of *PDS5B* exon1 to *STAG2* exon 3–34. Coverage of the aligned reads is indicated. (C) Left: Circos plot showing somatic SVs of ALL10 with secondary translocation t(2;10)(q37.1;p14) detected by OGM (left). Right: Detailed view of patient optical map (blue) partly aligning to reference chromosomes (green) 2 and 10, indicating a translocation that leads to potential fusion of *SFMBT2* exon1-2 (gray arrow) to *DGKD* exon 4–30 (red arrow). Potential breakpoints are indicated in pink. (D) Validation of *SFMBT2::DGKD* expression in ALL10 by RNA-seq. Arriba output of RNA-sequencing data is shown indicating a t(2;10)(q37.1;p14) that leads to in-frame fusion of *SFMBT2* exon1 to *DGKD* exon 4–30. Coverage of the aligned reads is indicated. BCP-ALL = B-cell precursor acute lymphoblastic leukemia; OGM = optical genome mapping; SV = structural variants.

OGM detected a translocation t(X;13)(q25;q13.1) in an *ETV6*::*RUNX1* BCP-ALL (ALL29) leading to potential fusion of exon 1 of the *PBS5B* gene to exon 3–35 of *STAG2*, which both encode for proteins of the cohesion complex (Figure 7A). By long-read sequencing and RNA-seq, we could validate the fusion of *PDS5B::STAG2* on DNA and mRNA level in the leukemic sample (Figure 7B; Suppl. Figure S12A and S12B). In ALL10, OGM detected a translocation t(2;10)(q37.1;p14) that potentially leads to in-frame fusion of *SFMBT2* exon 1–2 and *DGKD* exon 4–30 (Figure 7C). The *SFMBT2::DGKD* fusion was confirmed by long-read sequencing and expression in the leukemic sample was verified by RNA-seq (Figure 7D; Suppl. Figure S12C and S12D). In addition, the *SFMBT2* gene locus was potentially disrupted by a translocation t(10;19)(p14;p13.3) in case ALL5, which might also alter the *UHRF1* gene located on 19p13.3. We further validated the fusion of *TPRG1::BRD8* in ALL3 by long-read sequencing (Suppl. Figure S12E and S12F) and confirmed the expression of *TDRD5::LPCAT2* in an HD BCP-ALL (ALL59) and *PEX1::FGFR1OP2* in an *ETV6::RUNX1* BCP-ALL (ALL10) by RNA-seq, respectively (Suppl. Figure S13).

**Figure 8.**
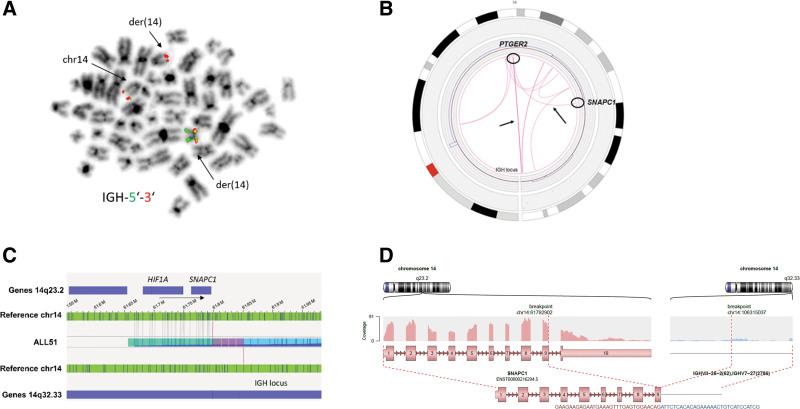
**OGM resolved complex *IGH* rearrangements in an HD leukemia patient.** (A) FISH result (with *IGH* 5′ and 3′ break-apart probes) is shown, indicating a t(14;14) with additional complex aberrations of the derivative chromosome 14 in ALL51. (B) Circos plot of chromosome 14 of ALL51 is depicted showing several intrachromosomal translocations including juxtaposition of the *IGH* region to the *PTGER2* loci and inversion of the *SNAPC1* region. (C) Optical map (blue) showing the juxtaposition of the *IGH* locus on 14q32.33 to the *SNAPC1* locus on 14q23.2. (D) Arriba output of RNA-sequencing data is shown indicating a t(14;14)(q23.2;p32.33) that leads to fusion of *SNAPC1* exon 1–9 to the *IGHVII-26-2/IGHV7-27* locus. Coverage of the aligned reads is indicated. HD = hyperdiploid; *IGH* = immunoglobulin heavy locus; OGM = optical genome mapping.

An immunoglobulin heavy locus (*IGH*) rearrangement was observed in an HD BCP-ALL case, ALL51, by FISH (using an *IGH* 5′ 3′ break-apart probe), which indicated the presence of a t(14;14) with complex additional aberrations on the derivative chromosome 14; without determination of the translocation partner (Figure 8A). By applying OGM, we found complex intrachromosomal translocations, which potentially lead to the juxtaposition of the *IGH* region on 14q32.33 to *SNAPC1*, likely via intrachromosomal translocation of the *IGH* locus to 14q22.1 (*PTGER2*) and concomitant inversion of the *SNPAC1* locus (Figure 8B and 8C). By RNA-seq, we could detect fusion of the *IGHVII-26-2/IGHV7-27* locus to *SNAPC1* (Figure 8D). However, no gene located on 14q was highly expressed, suggesting that the *IGH* translocation is rather a bystander product of the complex rearrangement on chromosome 14 than a stand-alone driver event in that particular case.

## DISCUSSION

By applying a combination of conventional cytogenetic and molecular genetic methods, WES and the novel OGM technique, we comprehensively resolved the landscapes of somatic SNVs/indels and SVs in 60 cases of the 2 most frequent subtypes of childhood BCP-ALL: *ETV6::RUNX1+* and classical HD. Our data adds to the molecular landscape of those subtypes determined by previous large-scale studies that applied SNP/micro-array and/or short-read NGS.^3,18–20,27–29^

In our study, OGM reliably detected 95% of SVs reported by molecular cytogenetics and SNP-array. In line with this, a high concordance of karyotyping and FISH in hematological malignancies has been shown in previous studies.^12,13,30–32^ By applying this new technique, we were able to detect an additional 677 SVs not identified by conventional techniques, of which 66% (448/677) were focal deletions with a median of 40.5 kb. These included 2 cryptic deletions within the highly repetitive region of the *IKZF1* gene^33^ that were missed by SNP-array likely due to poor probe coverage and low allele frequency. Other studies showed that OGM may become a particularly useful tool to identify patients belonging to the prognostically relevant *IKZF1*^*plus*^ group in a simple, fast, and cost-effective way.^12,17^ Of note, IKZF1^plus^ detection is usually assessed either by SNP-array or multiplex ligation-depended probe amplification,^25^ which are comparable in respect to detection limit and sensitivity of ≈20%. Consequently, *IKZF1* deletions may be difficult to detect in samples with low tumor cell content or leukemic subclones by conventional techniques. However, prognostic impact of subclonal deletions still needs to be evaluated in a clinical setting.^26^

Further, we could show that OGM is able to outperform Cyto-SNP12 array, depending on the resolution of the sensitivity of the array. OGM is a useful tool to further detect SVs in gene regions with probably low marker coverage in CytoScanHD-array, which is mainly enriched in known OMIM genes. Around 20% of leukemic blasts are required for SNP-array in a diagnostic setting for large CN alterations.^34,35^ Our study and others^14,36,37^ showed that OGM performance on samples with blast amounts of about 20%–30% could also detect alterations observed by conventional diagnostic methods. Hence, OGM could be an attractive option for routine diagnostic workup of hematological malignancies, as it has a fast turnaround time of ≈5 days from sample acquisition to data analysis.

We showed that the SV landscapes in *ETV6*::*RUNX1* and classical HD leukemia are profoundly different. Compared with HD BCP-ALL, *ETV6::RUNX1+* BCP-ALL harbored on average 3 times more SVs (mainly focal deletions) that might be associated with hyperactive RAG1/2 mediated recombination, which has been described as an oncogenic process in this specific subgroup.^38^ By contrast, there are limited analyses of aberrant RAG activity in HD BCP-ALL, and mechanisms of secondary SVs need to be further investigated. We further identified well-known large recurrent 12p deletions (≥5 Mb) in *ETV6*::*RUNX1* leukemia, which are associated with biallelic inactivation of the *ETV6* gene.^39^

By integrating OGM and WES, we identified 52 MARs affecting either 40 potential target genes or 12 intergenic regions. The latter regions are increasingly recognized in the field as possibly having functional effects on leukemia genomes through enhancer deletions, hijacking, and topically associated domain disruption, leading to gene dysregulation.^40–42^ Of note, we identified 4 intergenic MARs located in proximity (20 kb) of genes including 1q32.2 (−6 kb; *CD55*), 21q22.11 (−20 kb; *ATP5PO*), and 22q12.1 (−0.2 kb; *XBP1*). It should be noted that OGM has no exact breakpoint resolution and localization of MARs may have upstream and downstream variations dependent on label coverage of the identified region.

In line with previous findings, we identified SVs in both subgroups that affected several genes known to play a role in BCP-ALL development, including *ETV6, PAX5, BTG1, CDKN2A*, and *RAG2.*^3,38^ In addition to these well-described alterations, we identified SVs recurrently affecting *STAG2*, a member of the cohesin complex, including (1) 4 cases with focal intragenic deletions in the cohesin complex gene *STAG2*; (2) 1 case harboring a focal deletion within *STAG2* (16 kb, chrX:123,983,164–124,005,001 bp) and a concomitant missense mutation (p.Ile118Met); and (3) 1 case with a fusion of *STAG2* to *PDS5B* gene, which also encodes for a cohesin complex protein*. STAG2* inactivation has been implicated to cause aneuploidy in human cancer^43^; however, only 2 of the cases with intragenic *STAG2* deletion were HD tumors, and the 4 *ETV6*::*RUNX1* leukemia with *STAG2* alteration harbored no aneuploidy. The cohesin complex is involved in chromosome segregation in dividing cells, but is also implicated in more wide-ranging functions, such as DNA damage repair and regulation of gene expression, as indicated by the loss-of-function developmental syndrome, Cornelia de Lange.^44^ Previous studies based on the short-read sequencing, conventional technologies, and OGM reported somatic alterations of *STAG2* in BCP-ALL^3,17,38^ and showed that germline SNVs/indels in cohesin complex members, such as *NIPBL* or *RAD21,* could be involved in BCP-ALL predisposition.^45,46^ Consistently, studies in conditional knockout mice demonstrated that *Stag2* deletion in hematopoietic stem/progenitor cells increased their self-renewal capacity and impaired B-cell differentiation by decreasing chromatin accessibility and transcription of important B-cell transcription factors, such as PAX5.^47^ Our findings of recurrent *STAG2* alterations underlines the potential relevance of the cohesin complex genes to BCP-ALL development, arguably without involvement of chromosome segregation defects.

We further showed that a combined approach of OGM and NGS, including long-read and RNA-sequencing, is able to detect novel potential driver genes in BCP-ALL. Specifically, we were able to identify 19 recurrently altered regions in BCP-ALL with novel potential leukemic drivers. Twelve of these novel regions were not detected by SNP-array, which underscores the benefits of applying the OGM technique. We identified recurrently affected genes by SVs, such as *FOCAD*/*HACD4*, *MANBA*, and *SFMBT2* that were validated by long-read sequencing and/or RNA-seq in at least 1 leukemia. Recurrent potentially inactivating SVs in *SFMBT2* included the following: (1) an *ETV6::RUNX1*+ case (ALL10) with translocation t(2;10)(q37.1;p14), leading to fusion of the promotor region to *DGKD* and a concomitant loss of the whole *SFMBT2* gene; (2) an *ETV6::RUNX1+* case (ALL5) with a translocation t(10;19)(p14;p13.3) that might lead to disruption of *SFMBT2* and *UHRF1*; and (3) a HD case with an intragenic focal deletion (45 kb; chr10:7,286,100–7,343,440 bp), which hints at a potential role in leukemogenesis. *SFMBT2* is associated with histone-binding activity and negative regulation of gene expression.^48^ Its functional impact and role in BCP-ALL leukemogenesis should be addressed in the future.

We aimed to identify subtype-specific events of both genetic subgroups. In classical HD BCP-ALL, we did not find any recurrently affected region or gene by structural variation but SNVs/indels that recurrently affected genes including *KRAS*, *FLT3*, and *CREBBP*. In addition, *CREBBP* mutations are more prevalent in recurrent HD BCP-ALL.^24^ Consistently, one of the HD BCP-ALL relapses in our study already had a *CREBBP* mutation (p.Gln1491Lys) at the time of diagnosis, which is located in the HAT domain and was previously described in relapse prone HD BCP-ALL.^49^

In *ETV6*::*RUNX1*+ cases, we detected genes including *UHRF1* and *UBA2* recurrently altered by SV or SNV. Those genes are involved in sumoylation and ubiquitination, which is currently recognized as a new biological pathway in BCP-ALL,^3^ highlighting the importance of those pathways in *ETV6*::*RUNX1* leukemia. Interestingly, by using long-read sequencing, we detected that deletions (1) in ALL4 removed exon 1 and 2 of the *UHRF1* gene, which is coding for the ubiquitin homologues domain and (2) in ALL7 removed exon 13 of the *UBA2* gene, which is coding for the ubiquitin/SUMO-activating enzyme ubiquitin-like domain. A recent study further showed that a frameshift variant of *UBA2* was discussed to predispose to *ETV6::RUNX1*+ leukemia in monozygotic twins.^50^

Novel *ETV6::RUNX1* specific regions detected in our study encompassed genes such as *GPRC5A, MED13L*, and *MIB1* that might play a role in leukemogenesis. The MAR of 3 cases (chr18:21,727,621–21,759,839 bp) included the *MIB1* promotor region. *MIB1* encodes for an E3 ubiquitin-protein ligase that is involved in, among other processes, NOTCH signaling and ubiquitination of centriolar satellites.^51,52^ The *MIB1* gene locus has been shown to be hypomethylated in *ETV6::RUNX1+* BCP-ALL^53,54^ and is highly expressed in *ETV6*::*RUNX1* leukemia compared with other subtypes (MILE study, St. Jude Pecan Cloud).^55,56^ Interestingly, the relapsed sample of ALL4 harbored an acquired translocation t(17;18)(q12;q11.2) affecting the *MIB1* gene locus, indicating that deregulation of *MIB1* might play a role in leukemogenesis. Notably, we found 2 translocations and a stopgain mutation disrupting *MED13L* in 3 *ETV6*::*RUNX1*+ cases. *MED13L* is part of a mediator complex functioning as a coactivator for RNAII transcribed genes,^57^ and has been implicated in a case with constitutional haploinsuffiency in a syndromic patient who developed an ALL alongside clinical abnormalities.^58^

The identification of genes such as *ETV6, BTG1, STAG2, MANBA, TBL1XR1*, and *NSD2* that were affected by double hits of SVs and SNVs/indels demonstrates the power of combining OGM and WES for comprehensive determination of somatic alterations in BCP-ALL. Moreover, double hits affecting one target gene are indicative of a potential tumor suppressor^59^ that may have a strong relevance for ALL development. However, our study design with a combined approach of OGM and WES has a limited ability to detect compound heterozygosity due to lacking resolution on single-nucleotide level of OGM and short-reads surrounding the SNV. Only a scenario where for example a deletion overlaps with an SNV would suggest compound heterozygosity. That was not detected in any of the potential targets in our cohort. It is worth mentioning that secondary mutations in *TBL1XR1* have recently been implicated in improving risk stratification, especially in *ETV6*::*RUNX1* leukemia, where mutations led to inferior survival.^3^

Taken together, we have unraveled a profoundly different molecular make-up of *ETV6::RUNX1+* and classical HD BCP-ALL in terms of their landscape of structural aberrations. Moreover, we identified novel, recurrently altered genomic loci potentially involved in disease development and/or progression of these 2 BCP-ALL subtypes. Although OGM has obvious advantages compared with conventional technologies, comprehensive side-by-side evaluation and standardized round-robin tests must still be performed before it can be applied in routine diagnostics. And, only a fraction of genomic alterations (>500 bp) can be analyzed with OGM, so other techniques such as RNA- and long-read sequencing must be performed in parallel to cover the full spectrum of alterations. The rapidly developing third-generation high-fidelity long-read sequencing techniques might be advanced tools to decipher the whole spectrum of SVs and SNVs/indels in BCP-ALL and other tumor entities in the near future.

## ACKNOWLEDGMENTS

The authors thank Silke Furlan, Daniel Scholtyssik, and Katayoun Alemazkour for their excellent technical assistance. Computational support and infrastructure was provided by the Center for Information and Media Technology (ZIM) at the University of Dusseldorf (Germany). The authors greatly acknowledge the help of Bärbel Überlacker, Laboratory Zotz-Klimas, Dusseldorf. They thank Peter Ebert of the Core Unit Bioinformatics of the Medical Faculty of the Heinrich-Heine University for bioinformatic support.

## AUTHORS CONTRIBUTIONS

DB generated the molecular genetic data. DB and RW analyzed the molecular genetic data. AKB and KN provided cytogenetic and molecular cytogenetic data. SK, OAH, MS, TB, and AA provided patient samples and clinical data. LY and JA performed bioinformatic analysis of WES data. TM and JE processed long-read sequencing data. DS and DP provided bioinformatic analysis of RNA-seq data. UF, RW, and AB designed and supervised the project. All authors read and approved the final article.

## DISCLOSURES

The authors have no conflicts of interest to disclose.

## SOURCES OF FUNDING

DB was funded by the Dusseldorf School of Oncology (DSO, Medial Faculty of the Heinrich-Heine University Dusseldorf). RW was funded by the Research Commission of the Medial Faculty of the Heinrich-Heine University Dusseldorf. AB was supported by the Tour der Hoffnung, the Katharina-Hardt-Stiftung, the German Children’s Cancer Foundation, the German Federal Office for Radiation Protection (BfS, FKZ 3622S32231). UF was funded by the German Jose Carreras Foundation (DJCLS 18R/2021), the BfS (FKZ 3622S32231), the German Research Foundation (HE 8807/3-1), and the Cancer Prevention Graduate School, supported by the German Cancer Aid (grant no. 70114736) and coordinated by the German Cancer Research Center (DKFZ) (grant no. 70114766).

## Supplementary Material


