# Supplementary material for: Optical Genome Mapping Identifies Novel Recurrent Structural Alterations in Childhood ETV6::RUNX1+ and High Hyperdiploid Acute Lymphoblastic Leukemia
Source: Hemasphere. 2023 Jul 17;7(8):e925. doi: 10.1097/HS9.0000000000000925 (PMC10353714; doi:10.1097/HS9.0000000000000925)
Supplement: Supplementary file 2 [file hs9-7-e925-s002.docx]

**SDC, Material and Methods I**

**Bioinformatic processing, filtering and analysis of whole exome sequencing data**

Data was available for 33 non-tumor samples included in our previously published^1^ germline study and 26 non-tumor samples were newly sequenced. Somatically acquired SNVs/indels were detected using matched tumor and non-tumor samples from 58 patients. For two leukemia, single annotation was performed. Whole exome sequencing (WES) data was demultiplexed and trimmed using bcl2fastq and Trimmomatic.^2^ Reads were aligned to the hg38 reference genome applying the BWA mem algorithm.^3^ Five different callers were used in the default somatic mode (Varscan v.2.3.8^4^, Muse v1.0,^5^ Strelka v.2.9.1.0,^6^ Mutect2^7^ and LoFreq v.2^8^) to detect somatically acquired single nucleotide variants (SNVs) and short insertions and deletions (indels). Finally, all variant calls were merged and filtered with SomaticCombiner v1.02^9^ and annotation was performed using Ensembl Variant Effect Predictor v104^10^. We only considered somatic variants with a variant allele frequency ≥0.05 for further analysis. Damaging effects of recurrent SNVs/indels (n≥3 cases) were analyzed by *in silico* prediction tools (SIFT, PolyPhen, and CADD). Genes were only included in the oncoprint when harboring 1) truncating (frameshift, stopgain and/or splice-donor) variants and/or 2) SNVs/indels that were predicted to be damaging by at least two prediction tools. We further assessed pathogenicity by using the variant interpretation tool Franklin by Genoox (<https://franklin.genoox.com>).

**SDC, Material and Methods II**

**Detailed information on optical genome mapping (OGM) data filtering**

Somatic structural variants (SVs) were filtered using the following quality settings: 1) confidence score >0 for insertions and deletions; 2) confidence score >0.01 for inversions; 3) confidence score >0 for translocations; 4) confidence score -1 for duplications; 5) self-molecule count was set to 5; and 6) exclusion of B- and T-cell receptor rearrangements. The filtered lists of SVs were manually curated to exclude potential false-positives in, for example, centromeres or short acrocentric p-arms as well as to exclude SV duplets. Further filtering was performed for SVs only detected by the RVP algorithm. We excluded: 1) SVs with a variant allele frequency <0.05; 2) deletions/insertions <5 kb; 3) insertions >50 kb; 4) duplications <150 kb and 5) inversions <70 kb. For SVs detected only with the de novo assembly algorithm, we excluded deletions/insertions <1 kb. Copy number (CN) alterations were filtered using the following settings: 1) confidence score 0.99; 2) minimum length of 5 Mb; 3) fractional CN <1.7 for deletions; and 4) fractional CN > 2.8 for gains.

For downstream-analysis, we identified minimal altered regions occurring in at least three cases. For the oncoprint, we excluded regions where SVs were only detected by one calling algorithm and overlapped with common SVs of the dbVAR database^11^: 1) within OGM boundaries 2) with similar SV type and 3) with >70% size similarity.

**SDC, Material and Methods III**

**Detailed information on continuous long-read (CLR) sequencing analysis**

For the analysis in IGV, we extracted bam files of regions of interest to validate optical genome mapping (OGM) detected SVs. We detected deletions and translocations by analyzing split reads (at least one read) of the CLR data in the region of interest and their secondary alignment to estimate breakpoints. We concluded that OGM and CLR sequencing detected the same structural variant (SV) with following criteria: 1) for deletions, split reads must be located within the estimated OGM boundary of the respective SV and 2) estimated CLR breakpoints indicate size similarity >70% 3) for translocations, split reads must contain breakpoints located between OGM last matching label and closest nonmatching label.

**SDC, Material and Methods IV**

**Detailed information on RNA-seq workflow**

At the DKFZ NGS core facility, 500 ng total RNA of two samples (ALL10, ALL46) was processed using the TruSeq RNA Sample Preparation v2 kit (low-throughput protocol; Illumina, San Diego, CA, USA) and was sequenced using the NovaSeq6000 for 101 cycles. At the Genomics Transcriptomics Laboratory (BMFZ, Dusseldorf) two samples (ALL29, ALL59) were sequenced using the NextSeq2000 for 300 cycles. Fastq files of all samples were imported into Partek Flow (Partek Incorporated, St. Louis, MO, USA). Quality analysis and quality control were performed on all reads to assess read quality and to determine the amount of trimming required (both ends: 13 bases 5´ and 1 base 3´). Partek Flow default settings were used in all analyses.

**SDC, Figure 1**


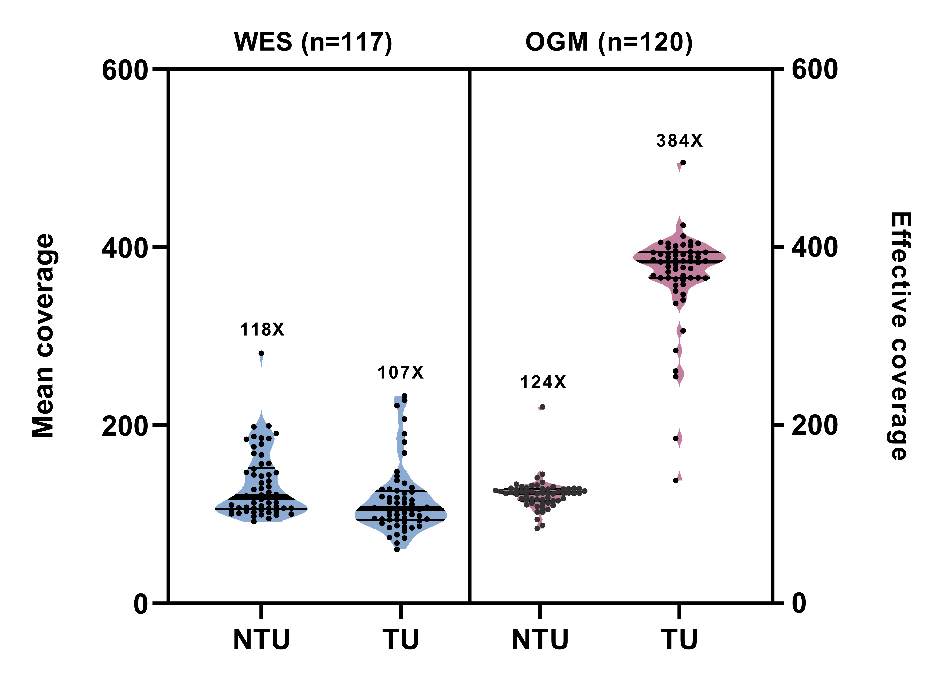


**SDC, Figure 1: Mean and effective coverage of WES (n=117 samples) and OGM (n=120 samples) data**. Each sample is depicted as a data point for matched non-tumor (NTU) and tumor (TU) samples. Violin plots show median (thick line), quartiles (slim line) and median is given in the figure. OGM= optical genome mapping; WES= whole exome sequencing

**SDC, Figure 2**

**
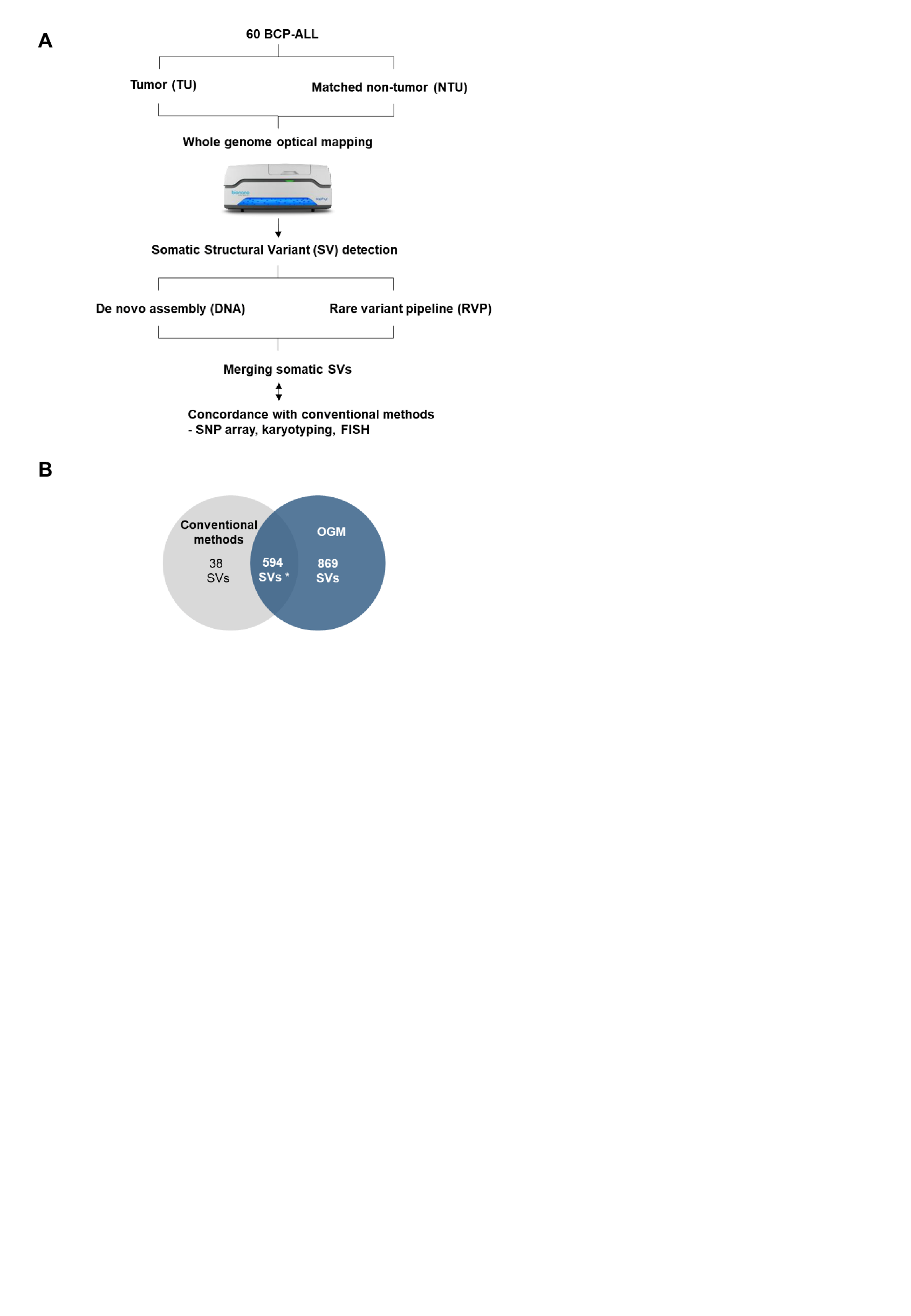
**

**SDC, Figure 2: Optical genome mapping workflow to detect somatically acquired SVs in 60 BCP-ALL.** Tumor and matched non-tumor samples were collected from 60 BCP-ALL cases. De novo assembly and rare variant pipeline were applied for SV calling using the duo-annotation mode allowing for the detection of somatic SVs. Filtered somatic SVs of each pipeline were merged to a final set of somatic SVs. Concordance of diagnostically reported SVs (karyotyping and FISH), SVs detected by SNP array and OGM was determined. OGM= optical genome mapping; WES= whole exome sequencing; SVs= structural variants; SNP= single nucleotide polymorphism; FISH= fluorescence in situ hybridization


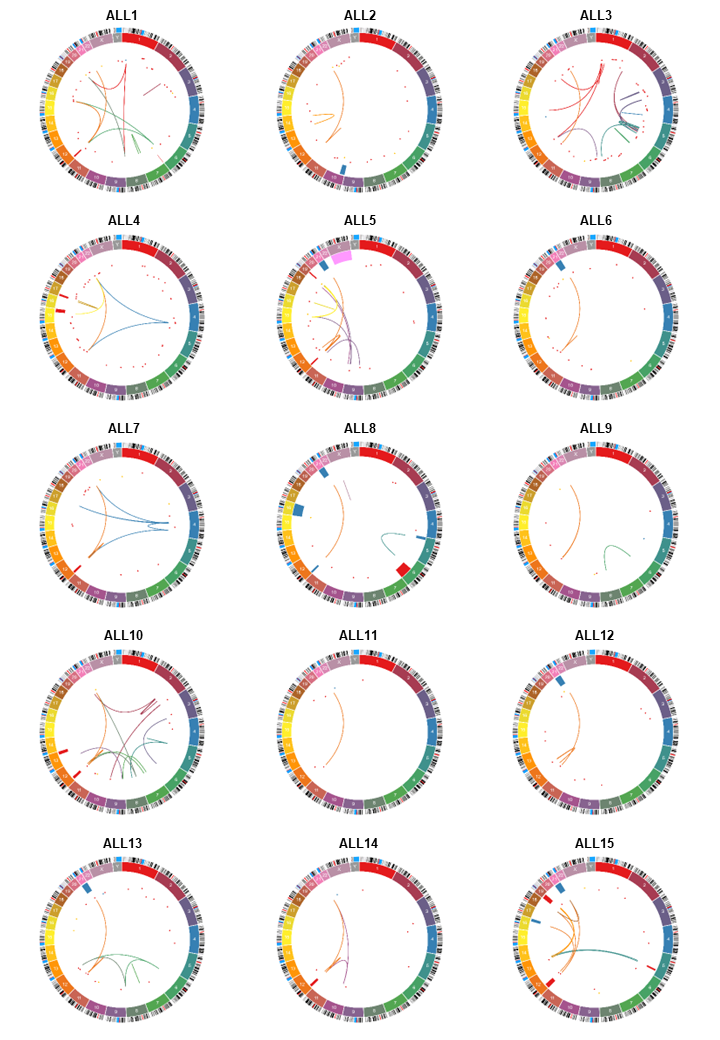
**SDC, Figure 3**
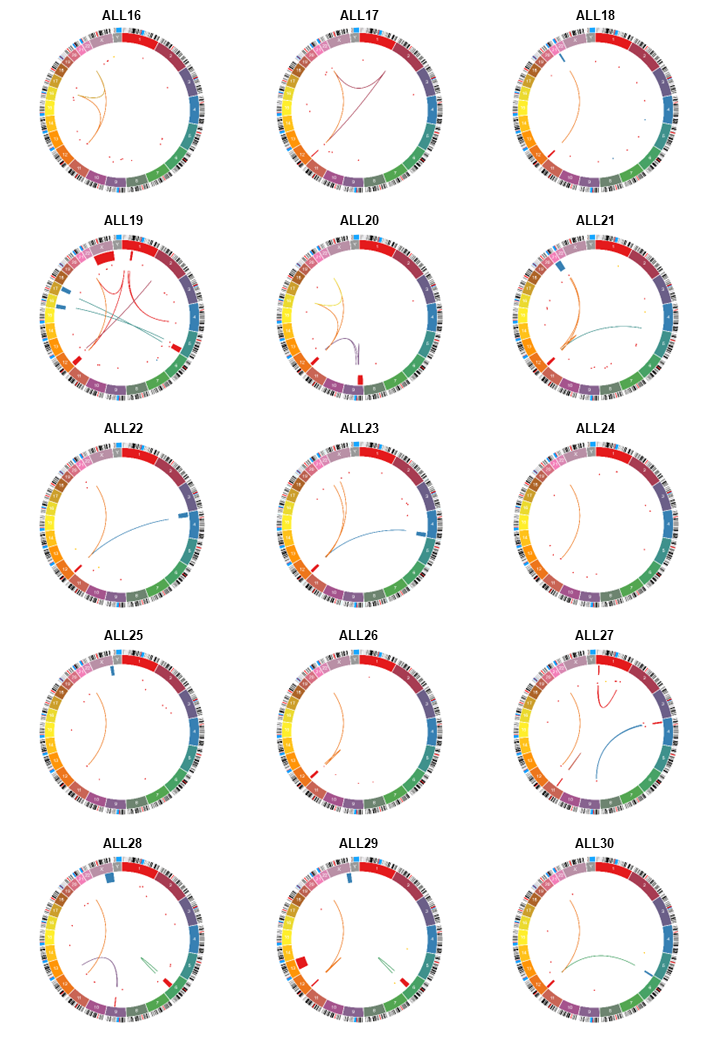


**SDC, Figure 3: Structural variation landscape of 30 ETV6::RUNX1 BCP-ALL detected by OGM and CNN-LOH detected by SNP array.** Circos plots of each ETV6::RUNX1 BCP-ALL case are shown. The circos plots depict from the outside to the inside circle: chromosomal ideograms, chromosomal numbers, SVs >5 Mb with duplications (blue), deletions (red) and CNN-LOH (pink); SVs <5 Mb with deletions (red), duplications (blue) and insertions (yellow) depicted as dots which indicate the start of the aberration. Inter and intrachromosomal translocations and inversions are indicated by lines connecting the proximate breakpoints. OGM= optical genome mapping; CNN-LOH= copy number neutral loss of heterozygosity; SNP= single nucleotide polymorphism; SVs= structural variants


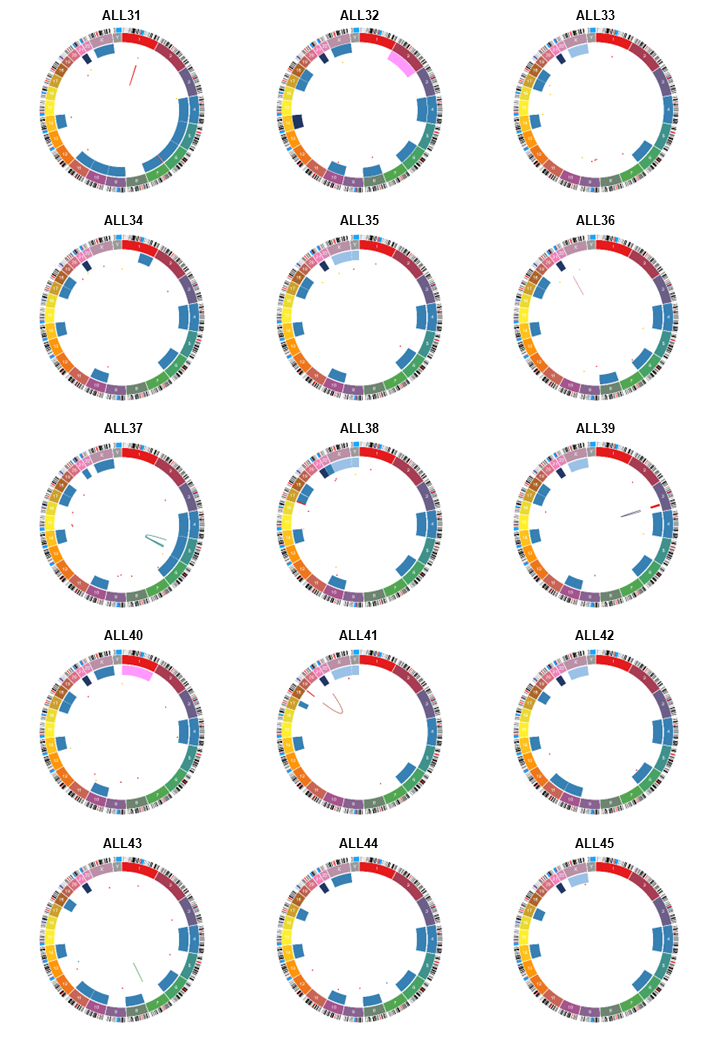
**SDC, Figure 4**


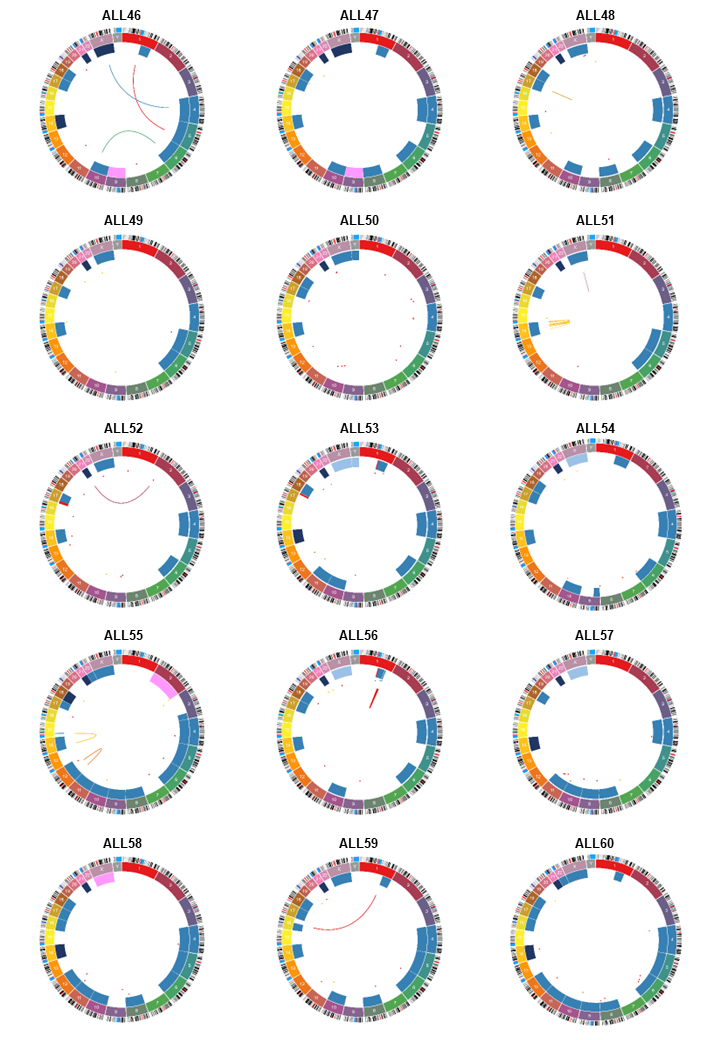


**SDC, Figure 4: Landscape of structural variants detected in 30 classical HD BCP-ALL by OGM and CNN-LOH by SNP array.** Circos plots of each HD BCP-ALL case are shown. The circos plots depict from the outside to the inside circle: chromosomal ideograms, chromosomal numbers, SVs>5Mb with duplications (blue), deletions (red) and CNN-LOH (pink); SVs <5Mb with deletions (red), duplications (blue) and insertions (yellow) depicted as dots which indicate the start of the aberration. Inter- and intrachromosomal translocations and inversions are indicated by lines connecting the proximate breakpoints of the corresponding chromosomes. OGM= optical genome mapping; HD= hyperdiploid BCP-ALL; CNN-LOH= copy number neutral loss of heterozygosity; SNP= single nucleotide polymorphism

**SDC, Figure 5**


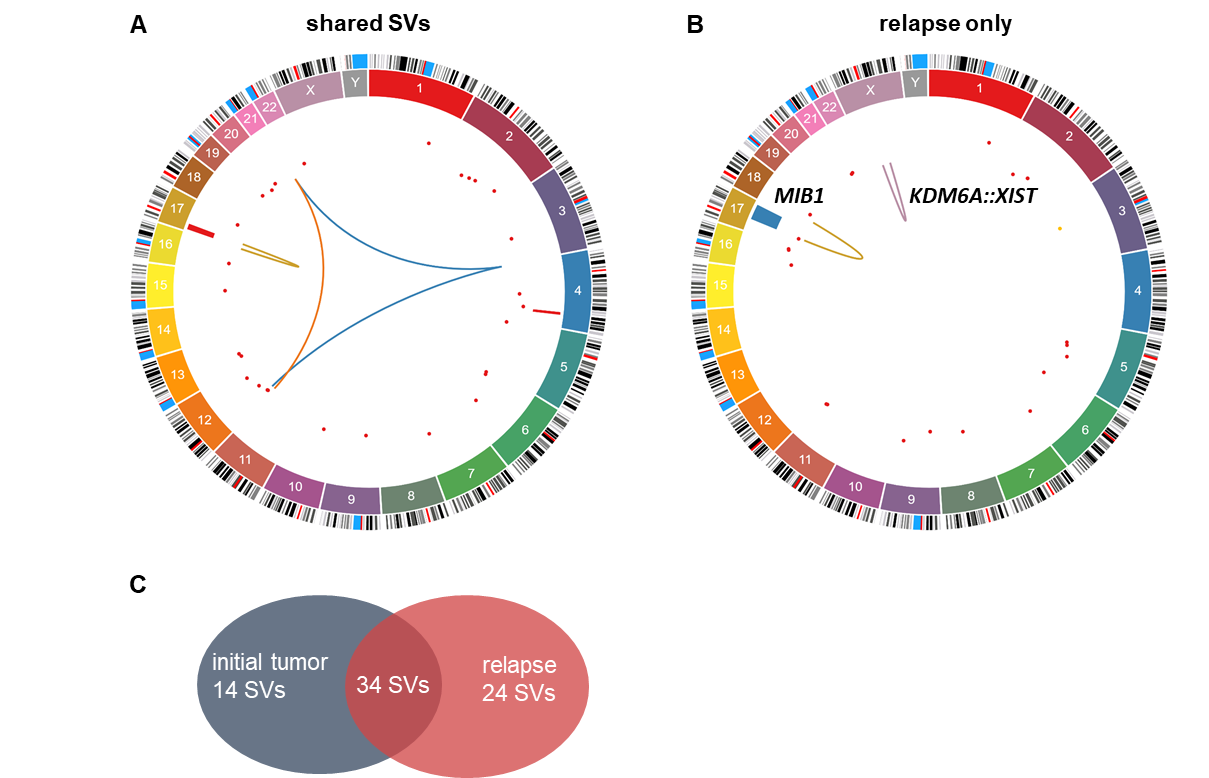


**SDC, Figure 5: Tumor evolution in ETV6::RUNX1 BCP-ALL case ALL4, who developed a relapse 48 months after initial diagnosis.** Circos plots showing (A) SVs shared by initial and relapsed BCP-ALL samples and (B) SVs unique to the relapse sample identified by OGM including t(17;18)(q12;q11.2) affecting the MIB1 gene locus and inv(X)(p11.3;q13.2) leading to potential fusion of KDM6A::XIST. (C) Quantity chart showing that 34 SVs were shared between initial and relapse tumor samples, whereas 14 SVs were unique to the initial tumor and 26 SVs were acquired during disease progression and solely identified in the relapsed sample. SVs= structural variants; OGM= optical genome mapping

**SDC, Figure 6**


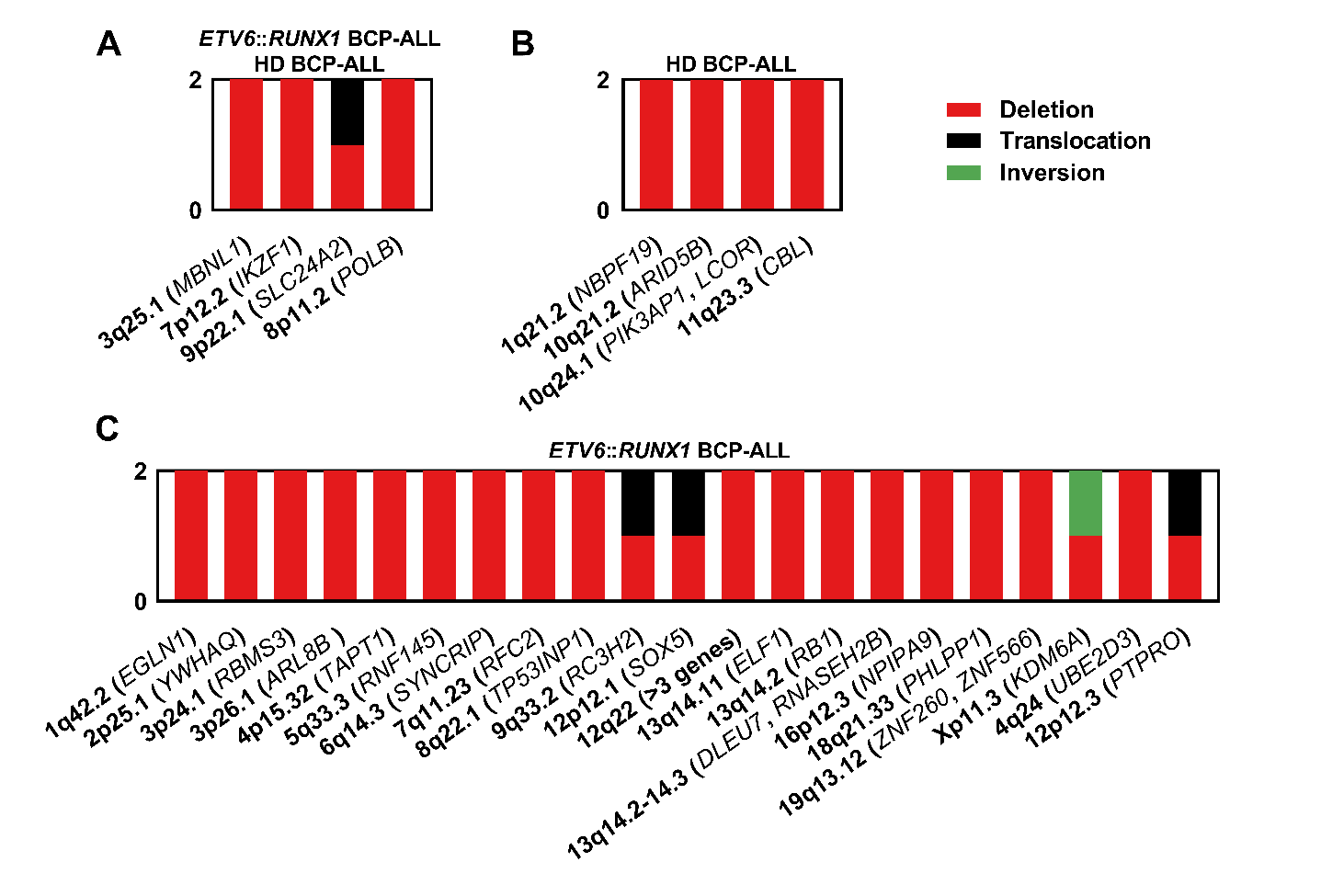


**SDC, Figure 6: Selected rare recurrently altered regions identified in 3% of BCP-ALL cases (n=2) by OGM.** Chromosomal regions and potential target genes are indicated. Rare recurrently altered regions detected in (A) ETV6::RUNX1 and HD BCP-ALL cases; (B) HD BCP-ALL cases; and (C) ETV6::RUNX1 BCP-ALL cases. OGM= optical genome mapping; HD= hyperdiploid BCP-ALL

**SDC, Figure 7
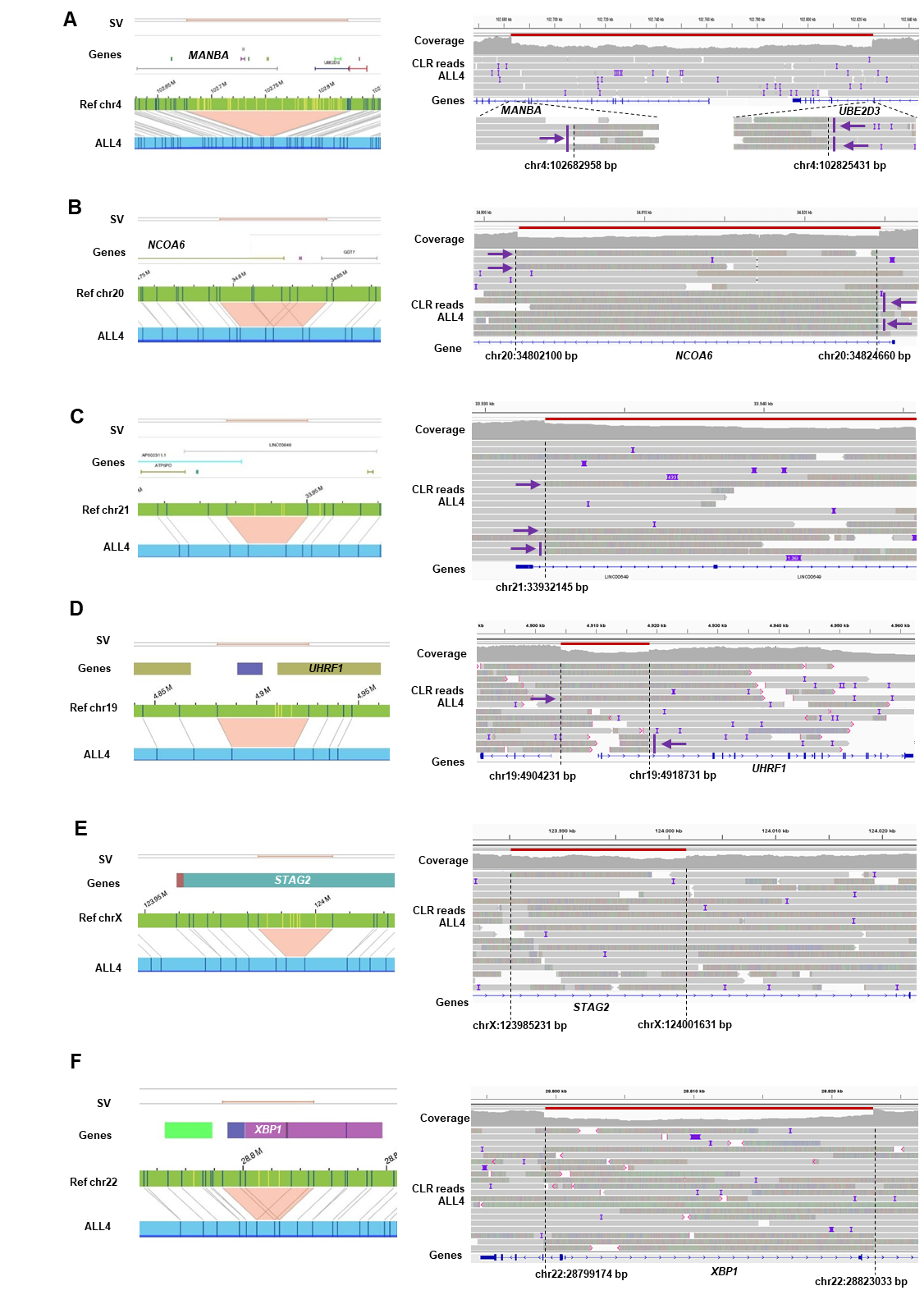
**

*
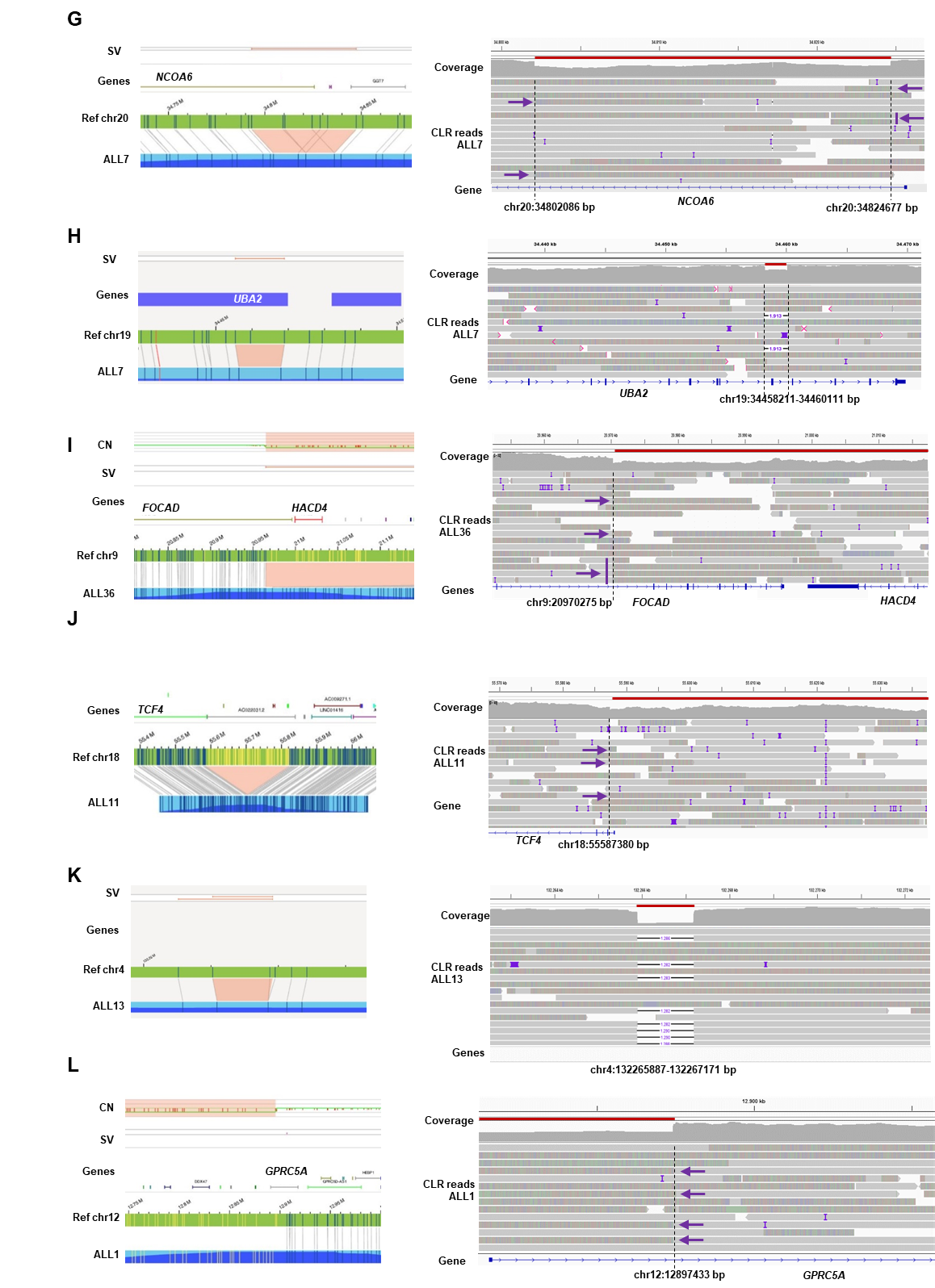
*


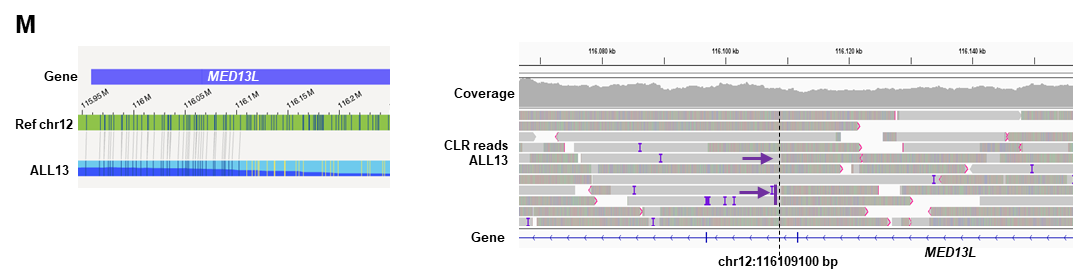


**SDC, Figure 7: Selected SVs detected by OGM and cross-validation with long-read sequencing in seven BCP-ALL.** Left: Optical map of the leukemia (blue) and respective reference map (green) indicating SV and overlapping gene. Red highlight indicates deletions. Split maps with aligned blue labels and unaligned yellow labels indicate translocations. Right: Continuous long-reads (CLR) aligning to the respective gene locus are shown (gray bars). Dashed lines indicate location of breakpoints. Split reads supporting the respective SV are marked by purple arrows. Decreased coverage of aligned reads support a deletion (red bar). (A) ALL4; 4q24 (MANBA) (B) ALL4; 20q11.22 (NCOA6) (C) ALL4; 21q22.11 (D) ALL4; 19p13.3 (UHRF1) (E) ALL4; Xq25 (STAG2) (F) ALL4, 22q12.1 (XBP1) (G) ALL7; 20q11.22 (NCOA6) (H) ALL7; 19q13.11 (UBA2) (I) ALL36; 9p21.3 (FOCAD; HACD4) (J) ALL11; 18q21.2 (TCF4) (K) ALL13; 4q28.3 (L) ALL1; 12p13.1 (GPRC5A) and (M) ALL13; 12q24.21 (MED13L). SVs= structural variants; OGM= optical genome mapping; CLR: continuous long-reads; Ref= reference

**SDC, Figure 8**

**
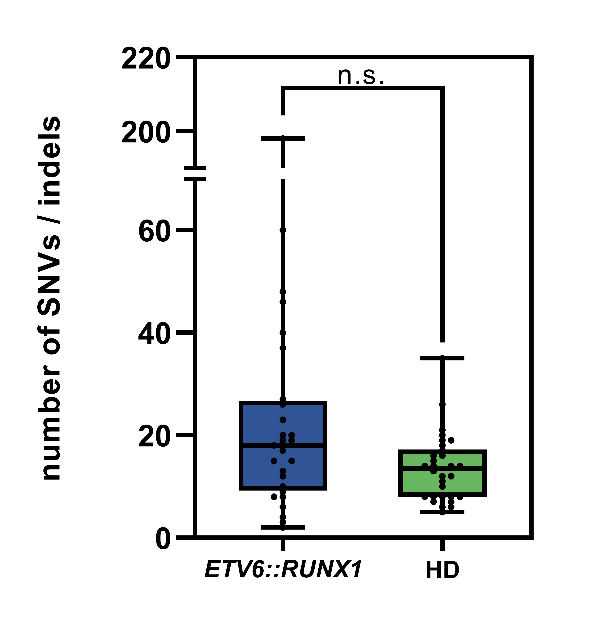
**

**SDC, Figure 8: Number of SNVs/indels detected in *ETV*6::*RUNX1* and HD BCP-ALL by WES.** Box plots showing number of SNVs/indels per case. Line, box and whiskers show median, quartile and +/- range. Statistical significance was assessed between the two subtypes using Mann Whitney U Test. n.s.= not significant; WES= whole exome sequencing; HD= hyperdiploid BCP-ALL; SNVs= single nucleotide variants; indels= insertions-deletions

**SDC, Figure 9**

**SDC, Figure 9:** Recurrently mutated genes (n≥3) identified by WES in 58 primary BCP-ALL. The most frequently mutated genes were *NRAS* (14/58, 24%), *NSD2* (6/58, 10%) and *ETV6* (5/58, 7%) in both BCP-ALL. HD BCP-ALL specific mutations were identified in *KRAS* (8/30, 27%), *CREBBP* (6/30, 20%), *FLT3* (5/30, 17%) and *PTPN11* (3/30, 10%). *ETV6*::*RUNX1*+ BCP-ALL subtype-specific mutations were observed in *CCDC168* (3/28, 11%) and *UBA2* (3/28, 11%). Each line indicates a gene and, each column a case. The color code reflects the mutation type. WES= whole exome sequencing; HD= hyperdiploid BCP-ALL

**SDC, Figure 10**


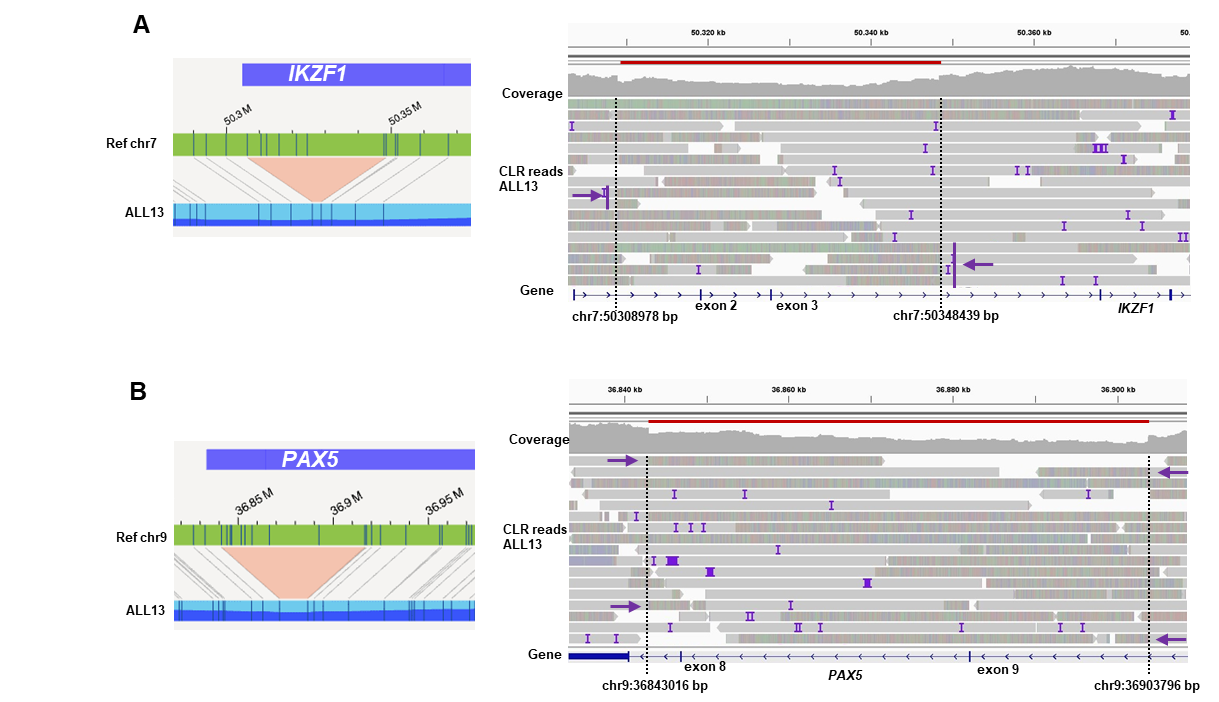


**SDC, Figure 10: High risk ETV6::RUNX1 BCP-ALL case harboring concomitant IKZF1 and PAX5 deletion.** (A) Left: Optical map of ALL13 (blue) compared to reference chr7 (green) indicating deletion in IKZF1. Right: ALL13 CLR reads mapping to the IKZF1 locus are shown. Split reads (purple arrows) and decreased coverage (red bar) reveal a deletion on chr7:50,308,978-50,348,439 bp, removing exon 2 and 3 of IKZF1. (B) Left: Optical map of ALL13 (blue) compared to reference chr9 (green) indicating deletion in PAX5. Right: ALL13 CLR reads mapping to the PAX5 locus are shown. Split reads (marked by purple arrows) and decreased coverage (red bar) reveal a deletion on chr9:36,843,016-36,903,796 bp, removing exon 8 and 9 of PAX5. CLR= continuous long-reads; Ref= reference

**SDC, Figure 11**


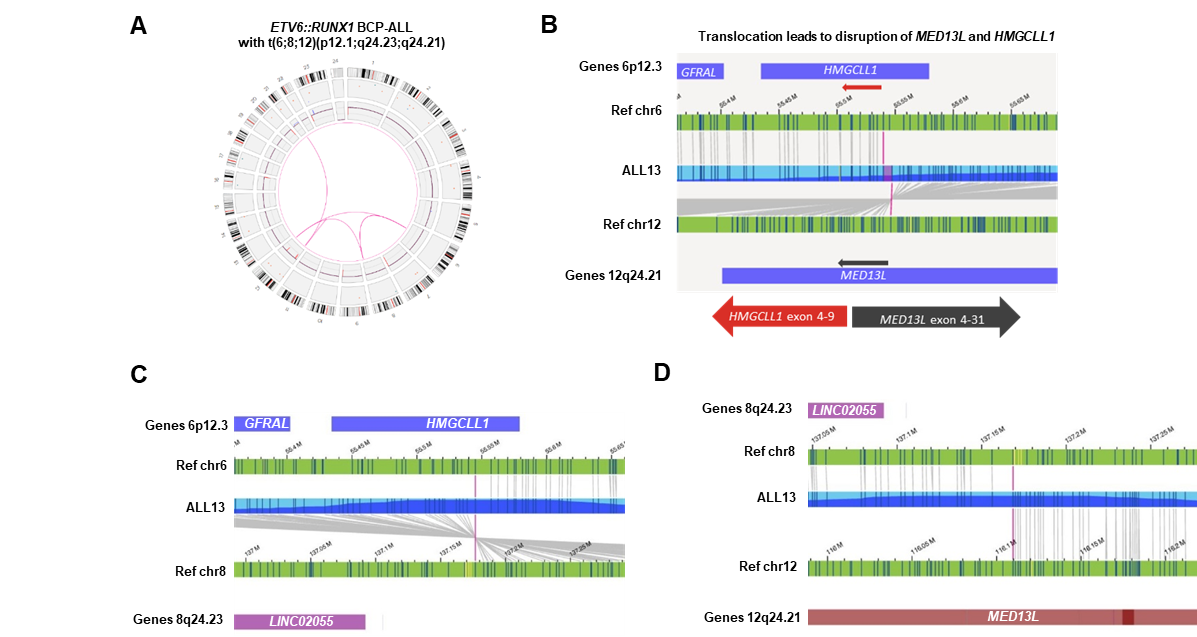


**SDC, Figure 11: ETV6::RUNX1 BCP-ALL with t(6;8;12)(p12.1;q24.23;q24.21).** (A) Circos plot showing secondary three-way translocation t(6;8;12)(p12.1;q24.23;q24.21) in addition to ETV6::RUNX1 translocation. (B), (C), (D) Detailed views of patient optical maps (blue) involved in the three-way translocation. Corresponding genes and their locations are indicated in the colored boxes (top, bottom) and maps of reference chromosomes are depicted in green. Potential breakpoints are indicated in pink. Ref= reference

**SDC, Figure 12**

**
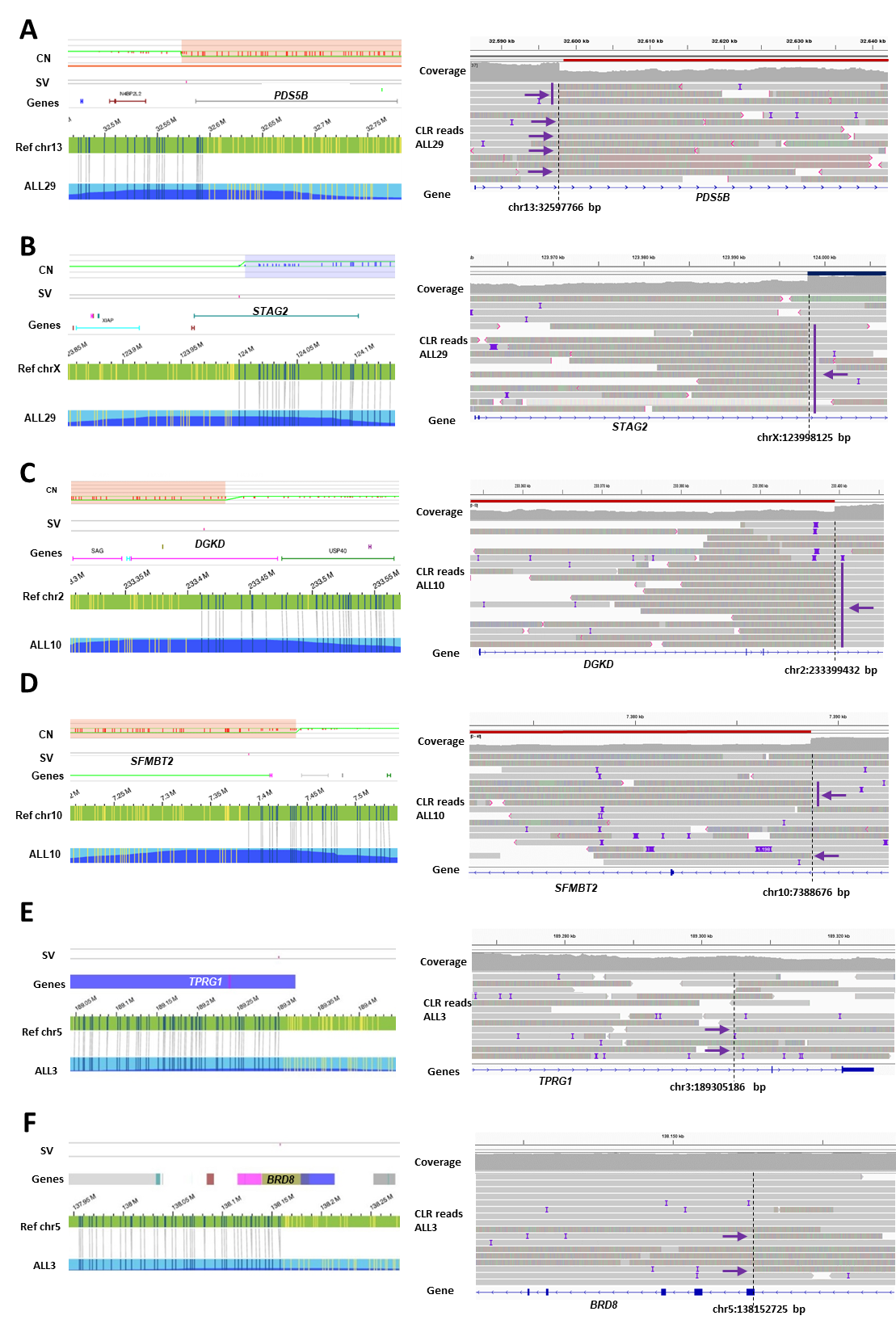
**

**SDC, Figure 12:**  **Cross-validation of potential in-frame fusion genes with long-read sequencing in three BCP-ALL.** Left: Optical map of the leukemia (blue) and respective reference map (green) indicating translocation and potential breakpoint in the overlapping gene. Split maps with aligned blue labels and unaligned yellow labels indicate translocations. Right: Continuous long-reads aligning to the respective gene locus are shown (gray bars). Dashed lines indicate location of breakpoints. Split reads supporting the respective translocation are marked by purple arrows. Decreased coverage of aligned reads support a deletion (red bar), increased coverage of aligned reads support a duplication (blue bar). (A), (B) t(X;13)(q25;q13.1) PDS5B::STAG2 (C), (D) t(2;10)(q37.1;p14) SFMBT2::DGKD and (E), (F) t(3;5)(q28;q31.2) TPGR1::BRD8. CLR= continuous long-reads; Ref= reference

**SDC, Figure 13**


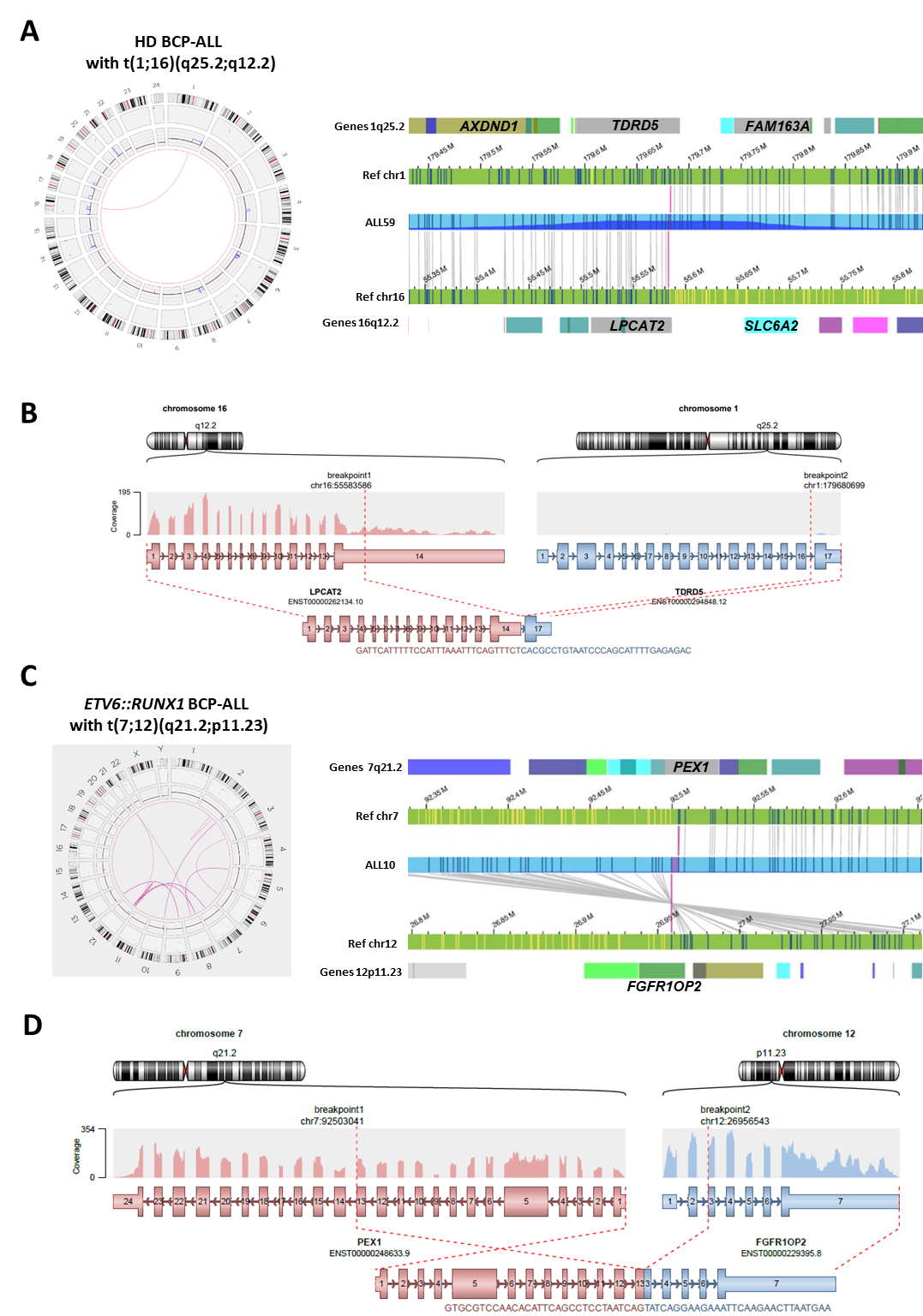


**SDC, Figure13: OGM and RNA-seq reveal novel fusion genes in *ETV6::RUNX1* and HD BCP-ALL.** (A) Left: Circos plot showing somatic SVs of an HD BCP-ALL (ALL59) with a secondary translocation t(1;6)(q25.2;q12.2) detected by OGM. Right: Patient optical map (blue) partly aligning to reference chromosomes (green) 1 and 16. Potential breakpoints (pink line) overlap with *TDRD5* and *LPCAT2*. (C) Validation of *TDRD5::LPCAT2* expression in ALL59 by RNA-seq. Arriba output of RNA-seq data is shown indicating t(1;16)(q25.1;q12.2) translocation leading to in-frame fusion of *LPCAT2* exon1-14 to *TDRD5* exon 17. Coverage of the aligned reads is indicated. (D) Left: Circos plot showing somatic SVs of an *ETV6::RUNX1* BCP-ALL (ALL10) with secondary translocations, including t(7;12)(q21.2;p11.23) detected by OGM. Right: Patient optical map (blue) partly aligning to reference chromosomes (green) 7 and 12. Potential breakpoints (pink line) overlap with *PEX1* and *FGFR1OP2*. (E) Validation of *PEX1::FGFR1OP2* expression in ALL10 by RNA-seq. Arriba output of RNA-seq data is shown indicating t(1;16)(q25.1;q12.2) translocation leading to in-frame fusion of *PEX1* exon1-13 to *FGFR1OP2* exon 3-7. Coverage of the aligned reads is indicated. OGM= optical genome mapping; HD= hyperdiploid BCP-ALL; SVs= structural variants

**SDC, References**

1. Wagener R, Taeubner J, Walter C, et al. Comprehensive germline-genomic and clinical profiling in 160 unselected children and adolescents with cancer. *Eur J Hum Genet*. Aug 2021;29(8):1301-1311. doi:10.1038/s41431-021-00878-x

2. Bolger AM, Lohse M, Usadel B. Trimmomatic: a flexible trimmer for Illumina sequence data. *Bioinformatics*. Aug 1 2014;30(15):2114-20. doi:10.1093/bioinformatics/btu170

3. Li H. Aligning sequence reads, clone sequences and assembly contigs with BWA-MEM. *arXiv preprint*. 2013;arXiv:1303.3997.

4. Koboldt DC, Larson DE, Wilson RK. Using VarScan 2 for Germline Variant Calling and Somatic Mutation Detection. *Curr Protoc Bioinformatics*. Dec 2013;44:15 4 1-17. doi:10.1002/0471250953.bi1504s44

5. Fan Y, Xi L, Hughes DS, et al. MuSE: accounting for tumor heterogeneity using a sample-specific error model improves sensitivity and specificity in mutation calling from sequencing data. *Genome Biol*. Aug 24 2016;17(1):178. doi:10.1186/s13059-016-1029-6

6. Saunders CT, Wong WS, Swamy S, Becq J, Murray LJ, Cheetham RK. Strelka: accurate somatic small-variant calling from sequenced tumor-normal sample pairs. *Bioinformatics*. Jul 15 2012;28(14):1811-7. doi:10.1093/bioinformatics/bts271

7. Benjamin D, Sato T, Cibulskis K, Getz G, Stewart C, Lichtenstein L. Calling Somatic SNVs and Indels with Mutect2. *bioRxiv*. 2019;02 Dec

8. Wilm A, Aw PP, Bertrand D, et al. LoFreq: a sequence-quality aware, ultra-sensitive variant caller for uncovering cell-population heterogeneity from high-throughput sequencing datasets. *Nucleic Acids Res*. Dec 2012;40(22):11189-201. doi:10.1093/nar/gks918

9. Wang M, Luo W, Jones K, et al. SomaticCombiner: improving the performance of somatic variant calling based on evaluation tests and a consensus approach. *Sci Rep*. Jul 30 2020;10(1):12898. doi:10.1038/s41598-020-69772-8

10. McLaren W, Gil L, Hunt SE, et al. The Ensembl Variant Effect Predictor. *Genome Biol*. Jun 6 2016;17(1):122. doi:10.1186/s13059-016-0974-4

11. Lappalainen I, Lopez J, Skipper L, et al. DbVar and DGVa: public archives for genomic structural variation. *Nucleic acids research*. Jan 2013;41(Database issue):D936-41. doi:10.1093/nar/gks1213
